# The isolated choice effect: An underlying psychological mechanism influencing racial diversity in organizations

**DOI:** 10.3389/fpsyg.2022.964959

**Published:** 2022-08-18

**Authors:** Hanyang Luo, Wanhua Zhou, Wugang Song

**Affiliations:** College of Management, Shenzhen University, Shenzhen, China

**Keywords:** isolated choice effect, variety seeking, racial diversity in organizations, combined and separated choices, workforce diversity, organizational psychology

## Abstract

With the view toward improving the racial diversity in organizations, this work seeks to uncover the reasons why larger groups have an advantage in terms of job opportunities. Based on people’s preference for diversity in commodity selection, we propose a potential feature that may exist in human resource management and call it the *isolated choice effect*, which unconsciously affects the racial diversity of organizations. Specifically, when making selections in isolation (i.e., when they are responsible for selecting a single person at a time), people are less likely to choose the one whose race would increase group diversity than when making selections in collections (i.e., when they are responsible for selecting several people at a time). We set up eight experiments (*n* = 2,792) in which participants make hiring or firing decisions among choices that are more white people than black people. We find that participants in the isolated choice group are less likely to choose black people, the smaller group, than those in the collective choice group. Our results show a potentially important contributing factor to the underrepresentation of black people in many organizations because hires are often made in isolation while layoffs are often made in collections, which provides a starting point for improving racial diversity in organizations by avoiding the isolated choice effect.

## Introduction

Racial diversity in organizations has been society’s constant concern in the United States. For example, Title VIII of the Civil Rights Act protects individuals against racial discrimination in employment, and the majority of Fortune 500 companies emphasize workplace diversity on their company homepages (Jones and Donnelly, 2017). In spite of this, the problem of the black people’s employment remains serious. Only four CEOs of the top 500 American companies and only three senators in the US Senate are black people. In the epidemic caused by the novel coronavirus (COVID-19), whites suffered an unemployment rate of 11% in 2020; for black people it is doubled (Lambert, 2020), which has led them to grave human rights disasters.

Notably, inclusiveness and diversity become increasingly important for corporate strategy in a world of a large movement of migrations. A McKinsey study published in 2015 found that companies with a diverse ethnic workforce perform at least 35% better than comparable firms with a homogeneous workforce (Hunt et al., 2015). Most of the prior research also suggests that the cultural diversity of a country’s labor force is a key factor in its innovation and competitiveness in the global economy (European Commission, 2003; Valantine and Collins, 2015; Nielsen et al., 2017). However, recent research has proposed a growing recognition that disparities in the representation of women and racial groups persist due to the lack of an equitable institutional culture (e.g., Ofili et al., 2021). To help institute new strategies to support organizational diversity in particular with the critical moment of the COVID-19 pandemic, our work aims to unveil some important consequences caused by an unobservable feature of personnel administration. To be specific, achieving the goal of diversity is often left to executives who frequently make hiring decisions in isolation (i.e., every employee is hired in a separate period), making it difficult to focus on each employee’s impact on organizational diversity. The idea can be explained in another way: when an HR makes hiring decisions, he or she forms a collection of all the selected people varying in gender, appearance, religion, and so forth; however, if there is only one vacancy, then the HR makes only one hiring decision so the collection includes only one person, who cannot be “diverse” by his or her own. Therefore, HRs in the latter situation are less likely to notice the diversity of the personnel aggregation formed. In light of this, minority groups unconsciously become vulnerable in job hunting. For example, when hiring decisions are made in isolation, the nurses in the hospital are probably all women if the majority of applicants are female for each selection. But when several people are hired at once, the inspection personnel may be aware of diversity and thus consider hiring male nurses to increase diversity. Similarly, if a high percentage of applicants are white (which is consistent with the real world), hiring in isolation may have an unintended impact on organizational diversity.

We are not the first to examine this phenomenon. Called *isolated choice effect* in the work of Chang et al. (2020), it is proven to influence gender diversity in personnel selections. The impact of the isolated choice effect on racial diversity in organizations is an overlooked, yet important, issue. Existing studies related to such kinds of effect mainly focus on consumers’ choice bracketing, which indicates that consumers select less diverse product bundles when making consumption decisions one at a time than simultaneously (Simonson, 1990; Simonson and Winer, 1992; Read and Loewenstein, 1995; Read et al., 2001). This phenomenon is attributed to people’s preference on commodity diversification portfolio for various factors when they are able to afford several goods at a time rather than only one. For example, they attempt to avoid the risk of uncertain forecasts of their future preferences (Simonson, 1990; Barbara and Lehmann, 1991); they desire excitement, novelty (Raju, 1980), and anticipated satiation (McAlister, 1982) and thus seek variety in consumption. Being a bit different, Chang et al. (2020) propose the mechanism that people rarely weigh group diversity in isolated choice decisions because diversity is essentially a group attribute and it is less tangible in isolated choice conditions. The mechanism is used to clarify why hiring managers construct less gender-diverse groups when being in the isolated choice condition (i.e., when they are responsible for choosing only one member for the group at a time) than when being in the collective choice condition (i.e., when they are responsible for choosing multiple members for the group at a time). However, whether the isolated choice effect accounts for low racial diversity in organizations is left undiscussed. Our study attempts to fill this gap by conducting four experiments through Amazon Mechanical Turk, and four in our community.

In order to illustrate why many well-disposed organizations remain predominantly white though they espouse commitments to racial justice, we focus on racial diversity to examine whether the isolated choice effect is one of the contributing factors. Therefore, our work is an extension of that of Chang et al. (2020). Nevertheless, because layoff is also an important factor in causing a low employment rate, we attach equal importance to the layoff scenarios. We propose that (1) managers have a lower probability to hire applicants whose race would increase the workforce diversity of the to-join list when selecting candidates individually (i.e., when they are in charge of the recruitment of only one member each time) than when selecting candidates collectively (i.e., when they are in charge of the recruitment of multiple group members simultaneously); (2) managers are more likely to fire employees whose race would increase the workforce diversity of the to-leave list when making layoff decisions collectively (i.e., when they are in charge of the firings of several members at a time) than individually (i.e., when they are in charge of the firings of only a single member each time).

Across eight experiments (*n* = 2,972), our hypotheses were largely supported and our work is expected to make important theoretical and practical contributions. This study provides a plausible explanation for low racial diversity in organizations, namely the isolated choice effect. Moreover, we show that the variety-seeking of consumer behavior theory can be applied to the implementation of personnel management in organizations.

We organize our paper as follows. In the next section “Literature Review,” we review the studies related to the isolated choice effect and put forward our hypotheses. We then conduct eight experiments (*n* = 2,972) to prove our hypotheses in section “Materials and methods.” In the last section, we discuss our findings that show the important implications of the isolated choice effect and provide advice to increase organizational diversity.

## Literature review

We review two research streams that inform our work, namely, workforce diversity, and the discrepancy of variety seeking between combined and separated choices.

### Workforce diversity management

It is widely recognized that diversity in the workplace brings wider knowledge, broader perspectives, and novel innovation to organizations (McLeod and Lobel, 1992). Diversity is important because it can be difficult to get innovative ideas from homogeneous groups of like-minded people with similar work attitudes (Yadav and Lenka, 2020). Teams of employees from different backgrounds and experiences can create various ideas and options that help companies easily develop successful products or services and boost business growth (Ng and Sears, 2012). From a broad perspective, diversity also contributes to organizational resilience, as diverse teams can quickly recover from and go through unexpected shocks (Vallejo et al., 2020). In consequence, workforce diversity highly corresponds with better performance when diversity is supported and differences are embraced in various sectors (e.g., Riccucci, 2002; Gonzalez and DeNisi, 2009; Dandala, 2018; Aguwa et al., 2020).

Most of the research on workforce diversity management focus on two questions: why is workforce diversity important and how to promote workforce diversity? The former investigates the positive effects of diversity from dimensions of gender (Richard et al., 2004; Gonzalez and DeNisi, 2009), ethnicity (McLeod and Lobel, 1992), age (Joshi and Roh, 2009; Kearney and Gebert, 2009), educational background (Webber and Donahue, 2001), etc. in both laboratory and field studies. The latter intends to develop ways to increase the number of minority groups in the workplace (Estape et al., 2018; Glazer et al., 2018). However, their research lack an examination of the mechanisms by which diversity is influenced. Simply asking HR to recruit more minority workers can lead to a real sense of injustice, leaving these disadvantaged groups potentially subject to alienation and unrecognized strengths in the organization. Against this backdrop, it’s urgent to figure out the reasons causing low organizational diversity other than the factor of the population proportion. Chang et al. (2020) firstly propose the underlying psychological mechanism whereby hiring managers pay less attention to how their selected candidates affect the group diversity in *isolated choice* conditions to interpret the low proportion of females in various jobs. In other words, the low representation of women in the workplace is partly because most decisions are made in isolation, making it difficult to notice the impact each hire will have on workforce diversity from a global perspective. Inspired by this, we consider that the isolated choice effect may not only exist in gender diversity but also affect racial diversity. Moreover, their experiment did not take into account the situation of personnel layoff, which we believe is also a point of concern for workplace diversity.

### The discrepancy of variety seeking between combined and separated choices

According to the classical economic model, it is widely accepted that consumers face a decline in the marginal rate of return on consumption (i.e., the benefit from every extra unit of good would decline) and therefore consumers’ utility curves are normally convex toward the origin of coordinates, indicating that diversification provides consumers with higher utility. Much prior work also provide evidence that consumers prefer variety in their consumption bundles in order to meet their future needs (Lattin, 1987; Rozin and Markwith, 1991; Garg et al., 2007). A consumer may be satisfied with the attributes of a particular good, which will increase the attractiveness of offering alternatives to other attributes (McAlister, 1979) because people’s need for novelty, change, and complexity suggest the decreasing marginal value of choosing the same item (Driver and Streufert, 1965; Cummings and Venkatesan, 1976). This occurs when consumers purchase several goods simultaneously. However, Read and Loewenstein (1995) reveal that consumers who make purchases sequentially (i.e., on a series of separate occasions) rather than make purchasing decisions on several occasions at a time are less likely to select items with different attributes. The idea is the same as the isolated choice effect that people construct more gender-diverse groups when making sets of hiring selections than making isolated selection choices.

Our study follows the insights of Chang et al. (2020) who proved that variety-seeking exists in personnel selections in terms of gender diversity despite the important differences between purchasing behaviors and organizational hiring behaviors. Our work differs from them in three aspects: first, they only explore the effect of the isolated choice effect on gender diversity, whereas we focus on the effect of this mechanism on the opportunities for white people and black people to be selected in the workplace. Second, they only consider the case of hiring, whereas this paper considers both hiring and layoffs, which are equally important for employment. Third, the participants in the isolated condition of their experiments were different individuals, so the choices were not truly separated in time. The settings of our last four experiments intend to address the issue. Considering that extant studies have not examined the isolated choice effect in terms of racial diversity, we hypothesize:

*H1: Managers are less likely to hire applicants whose race would increase the diversity of the to-join list when making personnel selection choices in isolation than when making collections of choices.*

Based on the same logic, when a manager is asked to make firing decisions at a separate period (fire only one at a time), the set of people fired would be less diverse than when asking him or her to fire several people at a time. We thus hypothesize:

*H2: Managers are less likely to fire employees whose race would increase the diversity of the to-leave list when making personnel layoff decisions in isolation than when making collections of choices.*

## Materials and methods

To test our hypotheses, we designed eight experiments (*n* = 2,972). The experimental methods are questionnaire-based and are conducted by randomly distributing questionnaires on Amazon Mechanical Turk and the Wenjuanxing platform. The experiments ask participants to choose one person from several options according to the contextual setting, such as imagining themselves as an HR and choosing one from several candidates. All options are more white people, which is in line with most practical situations. The 2 × 2 × 2 groups of experiments are set up based on gender factors, two contexts of hiring and firing, and whether or not the decisions participants make are truly separate in time. To be specific for the last factor, in the first four experiments, we intentionally recruit participants in the isolated choice condition four times as many as in the inverse case (the collective choice condition). Each decision in the isolated choice condition was made by a different person, and thus they were not “time-split decisions” in the sense. In the last four experiments, the number of participants in the two conditions was equal because decisions made in the isolated choice condition were truly separated in time.

All questionnaires are divided into four major sections, namely, demographic characteristics of the participant, trap questions, main questions, and the participant’s feedback.

To save space, we did not show the demographic section in the questionnaires of Supplementary material. The demographic characteristics of the participants are shown in Tables 1, 2. The first four experiments on Amazon Mechanical Turk have slightly more male than female participants, which may be related to the predominance of male users on the platform. Since we favor participants with work experience, we chose to post the questionnaire during local class time. However, we still collected questionnaires from some participants who were younger than 18 years old and were students by profession, and we speculate that they may be teenagers who dropped out of school to earn a living from Amazon Mechanical Turk or college students who earned extra money from it. The lower proportion of older people in the last four experiments is because the questionnaire platform is relatively new and has more young users. Merchants and service personnel make up the majority of participants.

**TABLE 1:** Demographic characteristics of participants (in numbers).

Demographic characteristics		1A	1B	2A	2B	3A	3B	4A	4B
Gender	Male	294	259	263	274	104	113	122	102
	Female	271	241	237	251	100	115	128	98
Age	<18	30	23	21	30	3	6	2	1
	18–25	224	206	212	226	113	135	139	121
	26–35	196	182	177	180	70	68	76	70
	36–45	67	52	50	51	15	11	23	7
	>45	48	37	40	38	3	8	10	1
Educational background	High school and below	102	97	92	88	25	21	33	18
	Undergraduate degree	377	338	349	361	146	166	192	127
	Graduate degree and above	86	65	59	76	33	41	25	55
Profession (multi-selection)	Student	81	68	70	56	52	62	59	51
	Unemployed	41	40	75	44	3	3	2	0
	Merchant	98	79	82	80	62	72	89	82
	Professional (teacher/lawyer/doctor)	12	3	6	6	15	7	7	11
	Service personnel (salesperson/server)	104	99	81	112	34	48	46	28
	Worker (factory worker/building worker)	28	18	7	12	3	1	5	5
	Company staff	45	39	46	43	28	30	29	17
	Freelancer (artist/musician/writer/kol)	152	144	126	159	0	2	1	1
	Civil servant	0	1	0	3	0	0	4	2
	Others	4	9	7	10	7	3	8	3
Working experience	<1 year	72	58	72	70	67	63	71	62
	1–3 years	218	200	177	195	79	101	95	89
	4–8 years	185	172	161	201	23	36	45	28
	>8 years	90	70	90	59	35	28	39	21

**TABLE 2:** Demographic characteristics of participants (%).

Demographic characteristics		1A	1B	2A	2B	3A	3B	4A	4B
Gender	Male	52.0%	51.8%	52.6%	52.2%	51.0%	49.6%	48.8%	51.0%
	Female	48.0%	48.2%	47.4%	47.8%	49.0%	50.4%	51.2%	49.0%
Age	<18	5.3%	4.6%	4.2%	5.7%	1.5%	2.6%	0.8%	0.5%
	18–25	39.6%	41.2%	42.4%	43.0%	55.4%	59.2%	55.6%	60.5%
	26–35	34.7%	36.4%	35.4%	34.3%	34.3%	29.8%	30.4%	35.0%
	36–45	11.9%	10.4%	10.0%	9.7%	7.4%	4.8%	9.2%	3.5%
	>45	8.5%	7.4%	8.0%	7.2%	1.5%	3.5%	4.0%	0.5%
Educational background	High school and below	18.1%	19.4%	18.4%	16.8%	12.3%	9.2%	13.2%	9.0%
	Undergraduate degree	66.7%	67.6%	69.8%	68.8%	71.6%	72.8%	76.8%	63.5%
	Graduate degree and above	15.2%	13.0%	11.8%	14.4%	16.1%	18.0%	10.0%	27.5%
Profession (multi-selection)	Student	14.3%	13.6%	14.0%	10.7%	25.5%	27.3%	23.6%	25.0%
	Unemployed	7.3%	8.0%	15.0%	8.4%	1.5%	1.3%	0.8%	0.0%
	Merchant	17.3%	15.8%	16.4%	15.2%	30.4%	31.6%	35.6%	41.0%
	Professional (teacher/lawyer/doctor)	2.1%	0.6%	1.2%	1.1%	7.4%	3.1%	2.8%	5.5%
	Service personnel (salesperson/server)	18.4%	19.8%	16.2%	21.3%	16.7%	21.1%	18.4%	14.0%
	Worker (factory worker/building worker)	5.0%	3.6%	1.4%	2.3%	14.7%	0.4%	2.0%	2,5%
	Company staff	8.0%	7.8%	9.2%	8.2%	13.7%	13.2%	11.6%	8.0%
	Freelancer (artist/musician/writer/kol)	26.9%	28.8%	25.2%	30.3%	0.0%	0.9%	0.4%	0.5%
	Civil servant	0.0%	0.2%	0.0%	0.6%	0.0%	0.0%	1.6%	1.0%
	Others	0.7%	1.8%	1.4%	1.9%	3.4%	1.3%	3.2%	1.5%
Working experience	<1 year	12.7%	11.6%	14.4%	13.3%	32.8%	27.6%	28.4%	31.0%
	1–3 years	38.6%	40.0%	35.4%	37.1%	38.7%	44.3%	38.0%	44.5%
	4–8 years	32.7%	34.4%	32.2%	38.3%	11.3%	15.8%	18.0%	14.0%
	>8 years	15.9%	14.0%	18.0%	11.2%	17.2%	12.3%	15.6%	10.5%

The section of trap questions is designed to weed out invalid questionnaires and inattentive participants. Trap questions focus on whether participants correctly understand the situation, such as whether they are making hiring or firing decisions, and how many decisions they need to make. Participants who failed to choose the right answers will not get paid accordingly. Participants in each group of experiments are divided into an isolated choice group and a collective choice group, and the number of the two groups needed to be corresponding. Therefore, when invalid questionnaires are found, we recruit additional participants to make up the specified number.

In the main questions of the questionnaires, participants were asked to select one option from each group of mixed black people and white people. We compared the differences between the two choice scenarios based on comparing the proportion of black people chosen by participants in the isolated choice group to those in the collective choice group. We predicted that the former group would be less likely to choose a black person because they would choose only one person, whereas the latter group would be more likely to be aware of diversity by choosing multiple people. We set up reasonable situations for participants that were as close to the reality of the situation as possible. We also set up an obscuring choice for the collective choice group questionnaire in order to keep the participants from knowing the true purpose of our experiment and to confound the results.

In the final open-ended question, participants were asked about the factors they considered in making their decision. We will discuss the feedback they gave in the Discussion section. Additional details of the experiment are shown in the following sections, and please refer to our Supplementary material for the content of the questionnaires.

### Study 1A

In Study 1A, we set a hypothetical personnel selection scenario for investigating the influence of the isolated choice effect on the racial diversity of people on the to-join list. We predicted that participants who are in charge of single recruitment (i.e., participants who were placed in an *isolated choice* condition) would choose a lower proportion of black people than participants who are in charge of recruitment of multiple positions (i.e., participants who were placed in a *collective choice* condition).

We recruited participants who met our criteria through Amazon Mechanical Turk. These participants are located in the United States, with an approval rate for all the other requesters’ tasks greater than 98% and a number of tasks approved greater than 100. Though Amazon Mechanical Turk is not available in China, we conducted the experiments by remotely controlling the computer of one of our friends in London. After excluding participants who were trapped in our filtering questions, 565 participants were left. In order to satisfy the experimental setting, the number of participants was controlled to be divisible by five. Among them, 113 participants who were assigned to the collective choice condition were paid $0.5. They were required to make five hiring decisions that took about 5 min to complete. And the rest 452 participants in the isolated choice condition were paid $0.1 because they made only one hiring decision that took about 1 min to complete.

Participants were asked to think of that they were headhunters of a foreign trading company. The company was trying to hire five suitable candidates to fill five different positions: Purchasing Specialist, Accountant, Area Sales Representative, Quality Inspector, and Administrative Assistant (Area Sales Representative was designed to obscure our study’s focus on racial diversity so the data acquired from this selection was not considered in our analysis). Participants were divided into two groups. Each of the 113 participants in the *collective choice* condition was responsible for choosing five candidates – one person to fill each role; on the other hand, participants in the *isolated choice* condition were evenly distributed to make hiring decisions for the four roles (113 participants for each; the choice of Area Sales Representative is not designed for the isolated choice group) — each of the 452 was responsible for choosing only one candidate. As a result, the numbers of selected candidates are balanced across conditions because the number of participants in the *isolated choice* condition was 452, which is four times as many as 113 in the *collective choice* condition.

Firstly, participants were shown the statements of the job they are in charge of (e.g., “A purchasing specialist is responsible for buying the high-quality goods at the lowest possible price and in the appropriate quantity”). After reading the job statements, they were asked to select one person among three candidates for each role (so that they were engaging in joint evaluation) according to their pictures (virtual human faces taken from generated photos), years of experience, and ages. Except for the Area Sales Representative which included three whites, all the others contain two white males and one black male candidate. And we varied candidate quality such that the black man always had a moderate amount of experience and a moderate year of age. (i.e., The black man always had fewer years of experience and ages than one of the white men and more years of experience and ages than the other.) In order to enable participants in the collective choice condition to make all the hiring decisions in a more simultaneous manner, we put the five options on a single screen. Please refer to our Supplementary material (pages 2–12) for details of the survey.

#### Results 1A

The relevant variable that we are interested in was whether a black male was selected. Because there is a decision for obscuring study focus, we do not account it in our analysis. In the case of collective choice, black people were selected by 25.9% of all the selections made; however, in the case of isolated choice, black people were selected by only 14.8% of all hiring decisions. To predict whether a black male was added to the to-join list for each decision, we ran an ordinary least square regression. Since our analytical unit was a single selection, each decision made by participants in the *isolated choice* condition was counted in the regression only once, while each decision made under the *collective choice* condition was counted four times. The regression result shows that hiring in isolated choice condition has a negative impact on the probability of choosing a black man (see Table 3, *b*_*isolated*−*hiring*−1*A*_ = −0.278, SE = 0.026, *p* < 0.001; 95%*CI*: −0.329, −0.226), thus supporting H1 that managers in the *isolated choice* condition (who are responsible for hiring only one candidate) selected a lower proportion of black people than in the *collective choice* condition (who are responsible for hiring several candidates each time).

**TABLE 3:** Study 1A regression results.

Dependent variables: black male hired	
Isolated choice condition	–0.278*** (0.026)
Intercept	0.425*** (0.015)
Observations R^2^	1356 0.077

This table shows the results of an ordinary least squares regression predicting whether a black male was selected in each hiring decision as a function of experimental condition. Robust standard errors clustered by participant are in parentheses.

***Denotes significance at the 5% level.

### Study 1B

Given gender differences may affect our findings, we used a similar recruitment scenario to Study 1A but reversed candidates’ gender in each option. This time, participants were responsible for jobs that were closer to our real life and experience: they were asked to select restaurant staff that are commonly seen in daily life.

We recruited participants who met our criteria same as Study 1A through Amazon Mechanical Turk. 500 participants were left after excluding those who were trapped in our filtering questions. Among them, 100 participants who were assigned to the *collective choice* condition were paid $0.5. They were required to make five hiring decisions that took about 5 min to complete. Correspondingly, the rest 400 participants in the *isolated choice* condition were paid $0.1 because they made only one hiring decision that took about 1 min to complete.

In the experiment setting, participants were the owner of a newly opened restaurant. The restaurant was trying to hire five suitable staff to fill five different positions: Dishwasher, Kitchen Assistant, and Senior Chef (Senior Chef was designed to obfuscate our study’s focus on racial diversity so the data acquired from this selection was not considered in our analysis), Waitress, Restaurant Receptionist. Again, participants were divided into two groups – each of the 100 in the *collective choice* condition was responsible for hiring five candidates and each of the 400 in the *isolated choice* condition was responsible for hiring only one candidate. The number of participants in the latter was four times as many as that of the former such that the conditions were evenly balanced.

In the beginning, job descriptions of each position were shown to participants in charge (e.g., Kitchen Assistant: A Kitchen Assistant is apprenticed to the Senior Chefs. The Kitchen Assistant sometimes cooks simple cuisines). And then they were asked to choose one person for each role based on a joint assessment of the three candidates according to their pictures, years of experience, and ages. With the exception of three whites for Senior Chef, there were two white women and one black woman for each of the other four positions. And we varied candidate quality as the method mentioned in Study 1A. Participants in the *collective choice* condition were shown the five options with no page break. Please refer to our Supplementary material (pages 13–23) for details of the surveys.

#### Results 1B

The relevant variable of interest in this study was whether a black female was selected. Because there is a decision for obscuring study focus, we do not account this in our analysis. In the case of collective choice, black people were selected by 24.5% of all hiring decisions; however, black people were selected by only 18.8% of all hiring decisions in the other case. We ran an ordinary least squares regression to predict whether a black female was chosen for each hiring decision. Our result indicates the negative effect of being in the isolated choice condition on people’s likelihood of hiring a black (see Table 4, *b*_*isolated*−*hiring*−1*B*_ = −0.219, SE = 0.028, *p* < 0.001; 95%*CI*: −0.274, −0.163), thus providing further evidence for H1 that managers in the *isolated choice* condition (who are responsible for hiring only one candidate) selected a lower proportion of black people than in the *collective choice* condition (who are responsible for hiring several candidates each time).

**TABLE 4:** Study 1B regression results.

Dependent variables: black female hired	
Isolated choice condition	–0.219*** (0.028)
Intercept	0.406*** (0.016)
Observations R^2^	1200 0.048

This table shows the results of an ordinary least squares regression predicting whether a black female was selected in each hiring decision as a function of experimental condition. Robust standard errors clustered by participant are in parentheses.

***Denotes significance at the 5% level.

### Study 2A

Considering that personnel layoffs are also essential for employment, we therefore examined whether the isolated choice effect influences racial diversity of the to-leave list when people make firing decisions. Specifically, we predicted that participants responsible for firing a single employee (i.e., those assigned to an *isolated choice* condition) would choose a lower proportion of black people than participants responsible for firing several employees at a time (i.e., those assigned to a *collective choice* condition). We use the word “choose” here because firing is an act of choosing someone to leave, just as hiring is an act of choosing someone to come in.

Five hundred qualified participants were recruited. Among them, 100 participants who were assigned to the *collective choice* condition were paid $0.5. They were required to make five firing decisions that took about 5 min to complete, and the rest 400 participants in the *isolated choice* condition were paid $0.1 because they made only one firing decision that took about 1 min to complete.

This time, participants were told that they were the personnel managers of a foreign trading company. In response to the economic downturn, they need to cut one employee for each of five positions: Purchasing Specialist, Accountant, Area Sales Representative, Quality Inspector, and Administrative Assistant (Area Sales Representative was designed to obscure our study’s focus on racial diversity so the data acquired from this selection was not considered in our analysis). Each of the 100 participants assigned to the *collective choice* condition was responsible for the layoffs for all five positions. While the remaining 400 were equally assigned to four positions except for Area Sales Representative, and each had only one firing decision to make.

Participants were shown in advance the same job descriptions as Study 1A, along with the same employees’ photos and ages. We replaced “years of experience” with “years working in the company.” Except for the Area Sales Representative which included three whites, all the others contain two white males and one black male candidate. And we varied employee quality such that the black man always had a moderate amount of working years and a moderate years of age. After reading the information, participants were asked to choose one from three employees for each position to be downsized. Participants in the *collective choice* condition were shown the five options with no page break. For details of the surveys, please refer to our Supplementary material (pages 24–34).

#### Results 2A

The dependent variable we were interested in was whether a black male was fired. We did not account the decision made for Area Sales Representative in our analysis. In the case of collective choice, black people were selected by 21.8% of all firing decisions; in the case of isolated choice, however, black people were selected by only 10.3% of all firing decisions. We ran an ordinary least squares regression to predict whether a black male was fired. The regression result shows that under the *isolated choice* condition, the effect on the likelihood of downsizing a black was significant (see Table 5, *b*_*isolated*−*firing*−2*A*_ = −0.364, *SE* = 0.026, *p* < 0.001; 95%*CI*: −0.416, −0.313), thus supporting H2 that managers in the *isolated choice* condition (who are responsible for firing only one employee) choose a lower proportion of black people than in the *collective choice* condition (who are responsible for firing several employees at a time) when downsizing.

**TABLE 5:** Study 2A regression results.

Dependent variables: black male fired	
Isolated choice condition	–0.364*** (0.026)
Intercept	0.455*** (0.015)
Observations R^2^	1260 0.133

This table shows the results of an ordinary least squares regression predicting whether a black male was selected in each firing decision as a function of experimental condition. Robust standard errors clustered by participant are in parentheses.

***Denotes significance at the 5% level.

### Study 2B

In Study 2B, we ran a conceptual replication of Study 2A to explore whether the isolated choice effect on personnel layoff works for females.

Five hundred and twenty-five participants who passed our filtering question were qualified. Among them, 105 participants who were assigned to the *collective choice* condition were paid $0.5. They were required to make five firing decisions that took about 5 min to complete. And the rest 420 participants in the *isolated choice* condition were paid $0.1 becxause they made only one firing decision that took about 1 min to complete.

Participants were asked to imagine that they were the owner of a restaurant. The restaurant was trying to reduce costs by cutting five employees — one person for each of five positions: Dishwasher, Kitchen Assistant, Senior Chef (Senior Chef was designed to obfuscate our study’s focus on racial diversity so the data acquired from this selection was not considered in our analysis), Waitress, and Restaurant Receptionist. Participants were divided into two groups — each of the 105 in the *collective choice* condition was responsible for the decruitment of five people and each of the 420 in the *isolated choice* condition was responsible for the decruitment of only one person. The numbers of participants in the two groups were assigned such that the conditions were evenly balanced.

Job descriptions of each position identical to Study 1B were shown to participants in charge. There were three employees for each position. Participants were then asked to select and fire an employee based on a joint assessment of the three employees’ photos, years of service at the restaurant, and age. With the exception of three whites for Senior Chef, there were two white women and one black woman for each of the other four positions. And we varied employee quality as the method mentioned in Study 2A. Participants in the *collective choice* condition were shown the five options with no page break. The surveys are available in our Supplementary material (pages 35–45).

#### Results 2B

The relevant variable that we are interested in was whether a black female was fired. Because there is a decision for obscuring study focus, we do not account it in our analysis. In the case of collective choice, black people were selected by 21.4% of all firing decisions; in the case of isolated choice, however, black people were selected by only 9% of all firing decisions. We ran an ordinary least squares regression to predict whether a black female was downsized for each decision. The regression result shows that firing in the *isolated choice* condition has a negative impact on the probability of choosing a black woman (see Table 6, *b*_*isolated*−*firing*−2*B*_ = −0.346, *SE* = 0.027, *p* < 0.001; 95%, *CI*: −0.399,−0.293), thus providing further evidence for H2 that managers in the *isolated choice* condition (who are responsible for firing only one employee) selected a lower proportion of black people than in the *collective choice* condition (who are responsible for firing several candidates each time) when downsizing.

**TABLE 6:** Study 2B regression results.

Dependent variables: black female fired	
Isolated choice condition	–0.346*** (0.027)
Intercept	0.449*** (0.016)
Observations R^2^	1200 0.119

This table shows the results of an ordinary least squares regression predicting whether a black female was selected in each firing decision as a function of experimental condition. Robust standard errors clustered by participant are in parentheses.

***Denotes significance at the 5% level.

### Study 3A

In the *isolated choice* condition of the above experiment, each participant was only responsible for selecting one person. But in reality, personnel administrations are often executed by the same manager who has to experience several times of *isolated choice* conditions. Therefore, in Study 3A, we referred to the snack experiment Simonson did in 1990 [11]. In his study, Simonson provided students with snack options over a 3-week period. Students who randomly chose one snack each week were significantly more likely to choose the same snack each time than students who chose three snacks at a time. We predicted that participants responsible for hiring several candidates but only one at a time (i.e., those assigned to an *isolated choice* condition) would construct a collection with a lower proportion of black people than participants responsible for hiring several candidates simultaneously (i.e., those assigned to a *collective choice* condition).

Because of the experiment setting, recruiting online participants requires much effort and resources, we conducted the following four experiments (Study 3A–Study 4B) in our community in China. We recruited 204 participants from our community, and evenly assigned them to two groups – 102 for the *collective choice* condition and 102 for the *isolated choice* condition. Participants in the collective choice group were paid 2 yuan to fill out a survey that took about 5 min to complete. However, participants in the isolated choice group were paid 15 yuan to fill out three surveys that were distributed in separate periods. They were asked to think of themselves as the interviewers of a research institution that was looking for some male researchers. There were three first-round interviews, each with five male applicants, all of whom have a chance to enter the final round. Among the five applicants in each interview, two of them were black people with moderate quality.

(a) Group 1 (collective choice condition)

Participants were required to choose one person for each interview. They were given only 5 min (so as to make it as simultaneous as possible) to guess the candidates most likely to enter the final round based on their appearance, age, and work experience. As a result, each participant chose three out of 15 people at a time. All options for the three interviews were on the same page.

(b) Group 2 (isolated choice condition)

Different from Group 1, participants in this group had a 3-days interval between each selection (interview). That is, they chose one candidate every 3 days. Please refer to our Supplementary material (pages 46–54) for the details of the surveys.

#### Results 3A

The relevant variable we were interested in was whether a black male was selected. In the case of collective choice, black people were selected by 45.1% of all hiring decisions; in the other case, however, black people were selected by only 31% of all hiring decisions. We ran an ordinary least squares regression to predict whether a black male was hired. The result shows that hiring in the *isolated choice* condition has a negative impact on the probability of choosing a black man (see Table 7, *b*_*isolated*−*hiring*−3*A*_=−0.224, *SE* = 0.043, *p* < 0.001; 95%*CI*:−0.309,−0.139), thus supporting H1 that managers in the *isolated choice* condition (who made hiring decisions sequentially) selected a lower proportion of black people than in the *collective choice* condition (who made multiple hiring decisions simultaneously).

**TABLE 7:** Study 3A regression results.

Dependent variables: black male hired	
Isolated choice condition	–0.224*** (0.043)
Intercept	0.689*** (0.027)
Observations R^2^	510 0.050

This table shows the results of an ordinary least squares regression predicting whether a black male was selected in each hiring decision as a function of experimental condition. Robust standard errors clustered by participant are in parentheses.

***Denotes significance at the 5% level.

### Study 3B

Considering that gender differences may affect our results, we used a similar recruitment scenario to Study 3A but reversed the gender.

We recruited 228 participants from our community and evenly assigned them to two groups – 114 for the *collective choice* condition and 114 for the *isolated choice* condition. Participants in the collective choice group were paid 2 yuan to fill out a survey at a time. However, participants in the isolated choice group were paid 15 yuan to fill out three surveys that were distributed in separate periods. Participants were asked to think of themselves as the interviewers for a cram school that was looking for some female teachers. Again, there were three first-round interviews, each with five female applicants, all of whom have a chance to enter the final round. Among the five applicants in each interview, two of them were black people with moderate quality.

(a) Group 1 (collective choice condition)

Participants were required to choose one candidate for each interview. They were given only 5 min to guess the candidates most likely to enter the final round based on their appearance, age, and work experience. As a result, each participant chose three out of 15 people at a time. All options for the three interviews were on the same page.

(b) Group 2 (isolated choice condition)

Different from Group 1, participants in this group had a three-day interval between each selection (interview). That is, they chose one candidate every three days. Our Supplementary material (pages 55–63) provide further details about the surveys.

#### Results 3B

Our dependent variable of interest was whether a black female was selected. In the case of collective choice, black people were selected by 43.9% of all hiring decisions; in the other case, however, black people were selected by only 29.2% of all hiring decisions. We ran an ordinary least squares regression to predict whether a black female was hired for each selection. The result shows that under the isolated choice condition, the effect on the likelihood of hiring a black people was significant (see Table 8, *b*_*isolated*−*hiring*−3*B*_ = −0.269, *SE* = 0.040, *p* < 0.001; 95%, *CI*:−0.348,−0.190), thus providing further evidence for H1 that managers in the *isolated choice* condition (who made hiring decisions sequentially) selected a lower proportion of black people than in the *collective choice* condition (who made multiple hiring decisions simultaneously).

**TABLE 8:** Study 3B regression results.

Dependent variables: black female hired	
Isolated choice condition	–0.269*** (0.040)
Intercept	0.708*** (0.026)
Observations R^2^	570 0.072

This table shows the results of an ordinary least squares regression predicting whether a black female was selected in each hiring decision as a function of experimental condition. Robust standard errors clustered by participant are in parentheses.

***Denotes significance at the 5%level.

### Study 4A

In Study 4A, we examined whether the isolated choice effect influences racial diversity if firing decisions are always made by the same people. We predicted that participants responsible for firing several candidates but only one at a time (i.e., those assigned to an *isolated choice* condition) would construct a to-leave list with a lower proportion of black people than participants responsible for firing several candidates simultaneously (i.e., those assigned to a *collective choice* condition).

We recruited 250 participants in our community and evenly assigned them to two groups — 125 for the *collective choice* condition and 125 for the *isolated choice* condition. Participants in the collective choice group were paid 2 yuan to fill out a survey. While participants in the isolated choice group were paid 15 yuan to fill out three surveys that were distributed in separate periods. Participants were asked to imagine that they were the personnel manager of a translation company that included 15 male interpreters. Due to COVID-19, they need to lay off workers to keep costs down. The 15 interpreters were divided into three groups of five. Among the five interpreters in each group, two of them were black people with moderate quality.

(a) Group 1 (collective choice condition)

Participants were required to fire one person for each group in 5 min based on their appearance, age, and work experience. As a result, each participant fired three out of 15 people at a time. All options for the three choices were in the same page.

(b) Group 2 (isolated choice condition)

Different from Group 1, participants in this group had a 3-day interval between each choice (downsizing). That is, they fired one person every 3 days. Please refer to our Supplementary material (pages 64–72) for the details of the surveys.

#### Results 4A

The variable we were interested in was whether a black male was fired. In the case of collective choice, black people were selected by 42.4% of all firing decisions; in the case of isolated choice, however, black people were selected by only 32.3% of all firing decisions. We ran an ordinary least squares regression to predict whether a black was selected for each firing decision. The regression result shows that under the isolated choice condition, the effect on the likelihood of downsizing a black employee was significant (see Table 9, *b*_*isolated*−*firing*−4*A*_ = −0.193, *SE* = 0.039, *p* < 0.001; 95%, *CI*: −0.271, −0.116), thus supporting H2 that managers in the *isolated choice* condition (who made firing decisions sequentially) fire a lower proportion of black people than in the *collective choice* condition (who made multiple firing decisions simultaneously).

**TABLE 9:** Study 4A regression results.

Dependent variables: black male fired	
Isolated choice condition	–0.193*** (0.039)
Intercept	0.677*** (0.025)
Observations R^2^	625 0.037

This table shows the results of an ordinary least squares regression predicting whether a black male was selected in each firing decision as a function of experimental condition. Robust standard errors clustered by participant are in parentheses.

***Denotes significance at the 5%level.

### Study 4B

Considering that gender differences may affect our results, we used a similar recruitment scenario to Study 4A but reversed the gender.

We recruited 200 participants in our community and evenly assigned them to two groups — 100 for each of the two conditions. Participants in the collective choice group were paid 2 yuan to fill out a survey. While participants in the isolated choice group were paid 15 yuan to fill out three surveys that were distributed in separate periods. Participants were asked to imagine that they were the personnel manager of a translation company that included 15 female interpreters. Due to COVID-19, they need to lay off workers to keep costs down. The 15 interpreters were divided into three groups of five. Among the five interpreters in each group, two of them were black people with moderate quality.

(a) Group 1 (collective choice condition)

Participants were required to fire one person for each group in 5 min based on their appearance, age, and work experience. As a result, each participant fired three out of 15 people at a time. All options for the three choices were on the same page.

(a) Group 2 (isolated choice condition)

Different from Group 1, participants in this group had a three-day interval between each choice (downsizing). That is, they fired one person every three days. For further details of the survey please refer to our Supplementary material (pages 73–81).

#### Results 4B

The relevant variable we were interested in was whether a black female was fired. In the case of collective choice, black people were selected by 43% of all firing decisions; in the case of isolated choice, however, black people were selected by only 35% of all firing decisions. We ran an ordinary least squares regression to predict whether a black was selected for each firing decision. The regression result shows that firing in the *isolated choice* condition has a negative impact on the probability of choosing a black woman (see Table 10, *b*_*isolated*−*firing*−4*B*_ = −0.125, *SE* = 0.044, *p* < 0.001; 95%, *CI*: −0.212,−0.038), thus providing further evidence for H2 that managers in the *isolated choice* condition (who made firing decisions sequentially) fire a lower proportion of black people than in the *collective choice* condition (who made multiple firing decisions simultaneously).

**TABLE 10:** Study 4B regression results.

Dependent variables: black female fired	
Isolated choice condition	–0.125*** (0.044)
Intercept	0.65*** (0.028)
Observations R^2^	500 0.015

This table shows the results of an ordinary least squares regression predicting whether a black female was selected in each firing decision as a function of experimental condition. Robust standard errors clustered by participant are in parentheses.

***Denotes significance at the 5% level.

## Discussion

We developed two hypotheses to understand the role of different choice conditions in influencing the racial diversity of organizations. Our hypotheses were largely supported and their close connection provides new insights into interpreting the low organizational racial diversity many companies face.

Across eight experiments (*n* = 2,972), we find that people select less race-diverse groups when making isolated choices (i.e., when hiring or firing a single person each time) than when making collective choices (i.e., when hiring or firing several people at a time). Both Study 1A and Study 1B demonstrate that when candidates are all male or all female, people are less likely to choose black people when asked to choose only one person. In practice, companies often do isolated choices when hiring, and even if they are interested in hiring several people for the same position, the interviews are always one-on-one. In this case, they attend less to how their selected candidate will affect the racial diversity of the group. But if they select several people at a time, they realize that the organization has fewer black people, thus increasing the likelihood of selecting black people. Both Study 2A and Study 2B show that people choose a greater proportion of black people in the case of collective layoffs when the members are all male or all female. In practice, mass layoffs often occur, which are almost collective choice conditions for personnel managers. If there are more whites in the company, more white people are likely to be laid off. But that’s when they notice the diversity of the members being laid off, thus increasing the percentage of black people. The results of Study 3A and Study 3B are consistent with the results of the first two studies. The difference is that the participants in the isolated choice made decisions every three days, which is more in line with the condition we defined. And in reality, HR hires at longer intervals, sometimes up to several months. Thus, even if the same person makes the hiring decision, he may be unable to notice the characteristics of the person he previously chose and is less likely to choose black people who would increase diversity in the organization. Similarly, both Study 4A and Study 4B are complementary to the third and fourth experiments. All participants in the isolated choice group make a layoff decision at intervals, not by a different person. Both settings yield more black people laid off by managers in the collective condition.

No zero-selected options are found for each study. Each option is set up with only a photograph, age, and work experience for participants. Black people are always of the medium age and have medium work experience, and we did not identify any appearance defects in each photo. Based on the participant feedback we collected in the open-ended questions, we find that participants in the isolated choice condition are more likely to select members based on work experience, while a significant number of participants in the collective choice condition mentioned appearance and skin color as factors to consider. It indicates that participants who choose multiple individuals at the same time are more likely to notice racial diversity. In reality, awareness is more likely to arise when managers interact face-to-face with candidates.

### Contributions and implications

We make important contributions to research on workforce diversity and variety seeking. Our work goes beyond prior management studies that focus on the outcomes of organizational performance, especially emphasizing the significance of diversity (Han et al., 2020; Lee and Kim, 2020; Sharma et al., 2020). Our findings have significant implications for the understanding of why, despite a constant emphasis on diversity in the organization, black employment rates are still far below justified. In practice, the implementation of recruitment is usually done by individual managers who normally make only one personnel decision at a time. That is, when hiring candidates, they often deal with *isolated choice* conditions that are less likely to select applicants whose race would increase the diversity of the group. Black people are therefore less likely to be selected in most hirings in which managers hardly pay attention to group diversity (because any one individual cannot be “diverse”). On the other hand, because of the large transaction costs involved in changing positions, organizations generally do not fire individuals unless that person is detrimental to the organization’s interests. Most of the layoffs are due to the economic crisis (e.g., economic depression brought by COVID-19) or the replacement of humans with artificial intelligence (e.g., jobs like supermarket cashiers, translators, and deliverymen). That is, personnel managers making firing decisions are always being placed in a *collective choice* condition that several leaving people are chosen simultaneously. In light of human’s ability to focus on whatever is different or unusual and to quickly and accurately recognize the salience of diversity (Phillips et al., 2018), they are able to notice the group diversity when making multiple decisions, and hence construct more race-diverse group by choosing more black people than if they have chosen in isolation. As a result, black people as the minority group in the United States are always at a disadvantage – they are facing both fewer job opportunities and a higher risk of dismissal. And this disadvantage may not be caused by racism or quality, but by managers falling into the trap of diversity.

Our findings suggest that organizations interested in instituting strategies to increase workforce diversity may consider having decision makers hire people collectively rather than in isolation. For instance, a company can hire several people in one recruitment rather than hire a single person every time. Or a relatively moderate approach is to hear applicants’ voices but not to see their faces in interviews, as is the case with some talent shows. On the black peoples’ account, our work suggests that applying to companies that hire several people at the same time has a better chance of being admitted.

Our results also provide new insights on variety seeking. The variety-seeking behavior of consumers is manifested as the conversion of brand product selection into the specific purchase behavior, which is a kind of consumption phenomenon opposite to repeated purchase (Li et al., 2007). In this process, the fundamental reason for consumers to seek diversification is not only the value brought by products and brands but also the utility brought by the switching behavior itself (Givon and Shapira, 1984). Chang et al. (2020) posit that managers, like consumers, tend to choose a diverse mix of the workforce as long as they notice diversity. We further validate their mechanism by replacing the gender variable in their work with the racial variable. And we show that though people cannot be consumed as goods, certain consumer behaviors can also be applied to organizational management.

Our study has some social implications. Our results show a potentially important contributing factor to the underrepresentation of black people in many organizations because hires are often made in isolation while layoffs are often made in collections. Normally people attribute the phenomenon to racial discrimination. There is a common belief that the personnel Manager has a color bias and is unwilling to implement policies that enhance diversity. The mere emphasis on increasing the number of black people makes white people feel unfair and does not really recognize black people’s abilities. People tend to think that a black person in an organization is chosen because his or her skin color could enhance diversity and contributes to the organization’s response to social responsibility, not because of the ability they value. Our research not only sheds light on why diversity is always so low, but it can also dispel some of the ideas in society that are wrongly perceived as “discrimination.” By adjusting the personnel management system to avoid the isolated choice effect, it can provide a fairer employment environment for both white people and black people, so that everyone’s ability can be recognized.

### Limitations and future work

Firstly, whereas we conducted experiments for both genders to enhance the robustness of our results, we did not design any choices which include people that are different in both race and gender. In reality, there may be black men and white women (or white men and black women) hunting for the same job. We thus suggest examining the isolated choice effect on group diversity with the interactions between gender and race.

Second, due to resource limitations, the last four experiments were conducted in China, where indigenous people have less contact with black people and white people. Future research can replicate our study to find out whether the experimental results in China differ from those in Europe and the United States.

Third, because our experiments are put in place in the same way as in the real world, there are always fewer black people than whites in the workplace. We therefore argue that in the case that all people are equally likely to be selected, there is a greater probability that the first person chosen by HR will be a white person. That is, black is always the race that would increase group diversity. If the HR continues to make the second choice (i.e., he or she is in a *collective choice* condition), he may notice the diversity and choose a black person and thus construct a more diverse group. But we have not actually measured this mechanism. It’s valuable for future research to test whether racial diversity is more salient in collective choice conditions than in isolated choice conditions and whether the salience of diversity mediates people’s hiring and firing decisions. For example, asking participants to what extent they consider their choice would influence the group diversity when making decisions. A moderation study is also meaningful to investigate whether it makes difference when drawing participants’ attention to racial diversity. For example, using a 2 × 2 (isolated choice versus collective choice × diversity valued versus control) factorial design by telling the diversity-valued group that “the organization strongly values diversity.”

In addition, according to demographic characteristics, some participants in the experiment worked for less than 1 year. They may lack experience in organizational personnel management and thus make choices that do not correspond to reality. However, we did not exclude such participants from the questionnaire due to limited resources. Future studies may consider recruiting only participants with longer work experience or even those who have worked as human resources recruiters, which is closer to reality.

## Conclusion

Our work empirically suggests the important role of the isolated choice effect in influencing organizational racial diversity. Whether it’s hiring or firing, people build more race-diverse groups when they have to choose several people at a time, rather than just one. Our work not only provides an important explanation for why some well-intentioned organizations remain remarkably homogeneous but also identifies potential solutions to address the problem.

## Data availability statement

The original contributions presented in this study are included in the article/Supplementary material, further inquiries can be directed to the corresponding author.

## Ethics statement

The studies involving human participants were reviewed and approved by the Academic Ethics Committee of Shenzhen University. The patients/participants provided their written informed consent to participate in this study.

## Author contributions

HL designed the study, analyzed the data, and reviewed and revised the manuscript. WZ proposed the research idea, designed the study, collected and analyzed the data, and wrote the manuscript. WS reviewed and revised the manuscript. All authors contributed to the article and approved the submitted version.
