# Supplementary material for: The isolated choice effect: An underlying psychological mechanism influencing racial diversity in organizations
Source: Front Psychol. 2022 Aug 18;13:964959. doi: 10.3389/fpsyg.2022.964959 (PMC9435528; doi:10.3389/fpsyg.2022.964959)
Supplement: Supplementary file 1 [file Data_Sheet_1.pdf]

Contents

Questionnaire 1 of Study 1A: Collective Choice condition 2
Questionnaire 2 of Study 1A: Isolated Choice condition 5
Questionnaire 3 of Study 1A: Isolated Choice condition 7
Questionnaire 4 of Study 1A: Isolated Choice condition 9
Questionnaire 5 of Study 1A: Isolated Choice condition 11
Questionnaire 1 of Study 1B: Collective Choice condition 13
Questionnaire 2 of Study 1B: Isolated Choice condition 16
Questionnaire 3 of Study 1B: Isolated Choice condition 18
Questionnaire 4 of Study 1B: Isolated Choice condition 20
Questionnaire 5 of Study 1B: Isolated Choice condition 22
Questionnaire 1 of Study 2A: Collective Choice condition 24
Questionnaire 2 of Study 2A: Isolated Choice condition 27
Questionnaire 3 of Study 2A: Isolated Choice condition 29
Questionnaire 4 of Study 2A: Isolated Choice condition 31
Questionnaire 5 of Study 2A: Isolated Choice condition 33
Questionnaire 1 of Study 2B: Collective Choice condition 35
Questionnaire 2 of Study 2B: Isolated Choice condition 38
Questionnaire 3 of Study 2B: Isolated Choice condition 40
Questionnaire 4 of Study 2B: Isolated Choice condition 42
Questionnaire 5 of Study 2B: Isolated Choice condition 44
Questionnaire 1 of Study 3A: Collective Choice condition 46
Questionnaire 2 of Study 3A: Isolated Choice condition 49
Questionnaire 3 of Study 3A: Isolated Choice condition 51
Questionnaire 4 of Study 3A: Isolated Choice condition 53
Questionnaire 1 of Study 3B: Collective Choice condition 55
Questionnaire 2 of Study 3B: Isolated Choice condition 58
Questionnaire 3 of Study 3B: Isolated Choice condition 60
Questionnaire 4 of Study 3B: Isolated Choice condition 62
Questionnaire 1 of Study 4A: Collective Choice condition 64
Questionnaire 2 of Study 4A: Isolated Choice condition 67
Questionnaire 3 of Study 4A: Isolated Choice condition 69
Questionnaire 4 of Study 4A: Isolated Choice condition 71
Questionnaire 1 of Study 4B: Collective Choice condition 73
Questionnaire 2 of Study 4B: Isolated Choice condition 76
Questionnaire 3 of Study 4B: Isolated Choice condition 78
Questionnaire 4 of Study 4B: Isolated Choice condition 80

(Questionnaire 1 of Study 1A: Collective Choice condition)

(The choices for collective choice conditions were shown in a single page in the questionnaires we presented to the participants)

In this study, you will imagine that you are a hiring manager for a foreign trading company. The company is currently trying to hire five suitable candidates to fill five different positions: Purchasing Specialist, Accountant, Area Sales Representative, Quality Inspector, Administrative Assistant.

You need to hire people to fill all five of these positions. It is your goal to hire the candidates whom you think will make the company the most successful.

Here are the job descriptions for each of the job titles:

Purchasing Specialist: A purchasing specialist is responsible for buying the high quality goods at the lowest possible price and in the appropriate quantity.

Accountant: An accountant maintains and audits business accounts for the company.

Area Sales Representative: An area sales representative is responsible for selling the products to consumers in the specific district and ensuring that consumers have positive experiences with the products.

Quality Inspector: A quality inspector is responsible for the quality inspection of materials and products.

Administrative Assistant: An administrative assistant provides various kinds of administrative support to organization members such as arranging meetings.

Q1. What's your goal in this survey?

A. Fire five employees
B. Evaluate consumer products
C. Hire five employees

Q2. There are three candidates for each of the positions. Please choose one from ABC.

Candidates for Purchasing Specialist

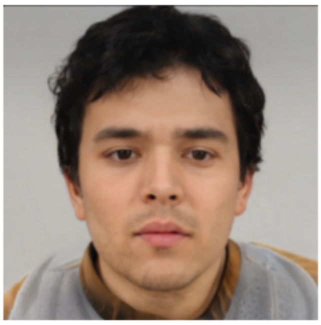

Age: 29
Years of experience: 5
A

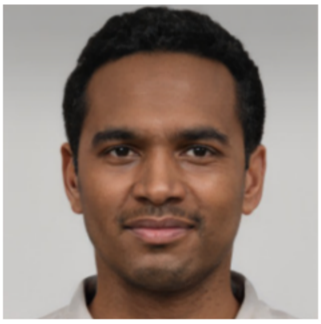

Age: 34
Years of experience: 2
B

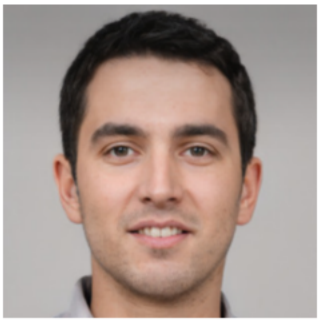

Age: 40
Years of experience: 0
C

Candidates for Accountant

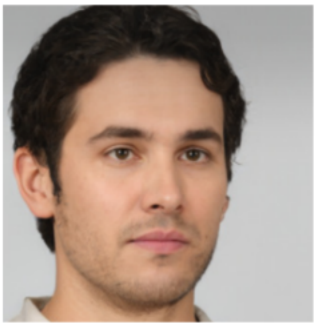

Age: 37
Years of experience: 11
A

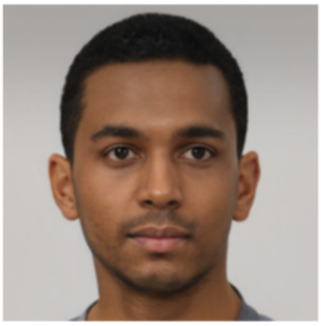

Age: 40
Years of experience: 8
B

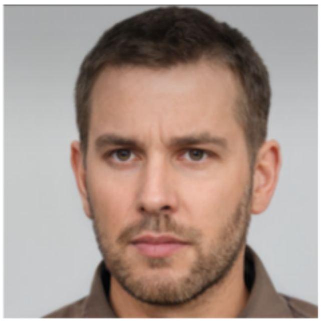

Age: 42
Years of experience: 7
C

Candidates for Area Sales Representative

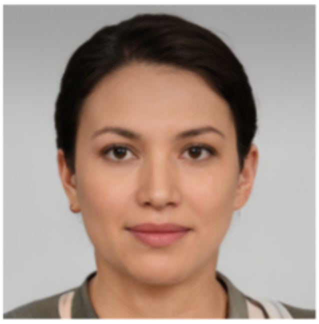

Age: 27
Years of experience: 4
A

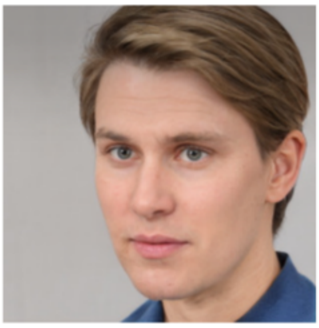

Age: 34
Years of experience: 2
B

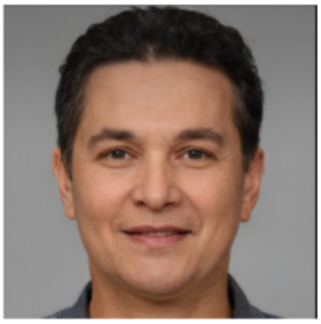

Age: 53
Years of experience: 8
C

Candidates for Quality Inspector

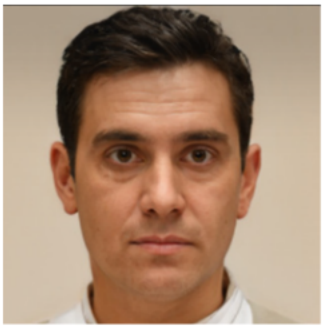

Age: 55
Years of experience: 6
A

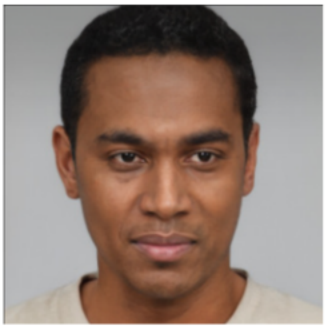

Age: 42
Years of experience: 4
B

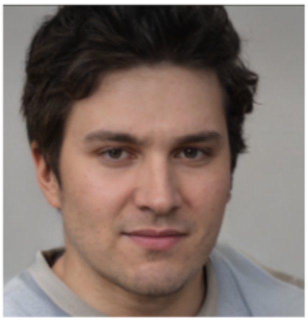

Age: 34
Years of experience: 0
C

Candidates for Administrative Assistant

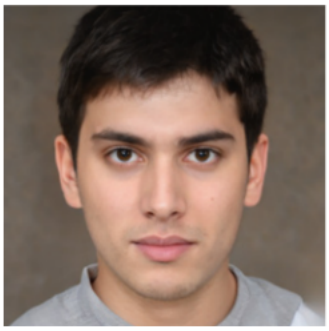

Age: 24
Years of experience: 1
A

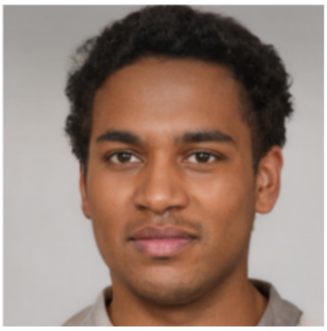

Age: 32
Years of experience: 2
B

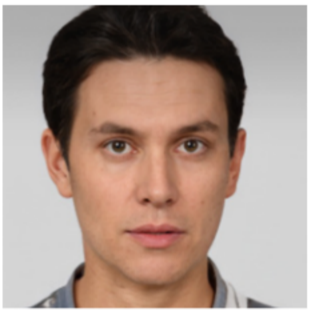

Age: 36
Years of experience: 3
C

Q3. What factors do you consider when choosing who to hire?

Q4. This space is provided if you have any additional feedback.

(Questionnaire 2 of Study 1A: Isolated Choice condition)

In this study, you will imagine that you are a hiring manager for a foreign trading company. The company is currently trying to hire a Purchasing Specialist.

It is your goal to hire the candidate whom you think will make the company the most successful.

Here is the job description for the job:

Purchasing Specialist: A purchasing specialist is responsible for buying the high quality goods at the lowest possible price and in the appropriate quantity.

Q1. What's your goal in this survey?

A. Fire a Purchasing Specialist

B. Evaluate consumer products

C. Hire a Purchasing Specialist

Q2. There are three candidates for this position. Please choose one form ABC.

Candidates for Purchasing Specialist

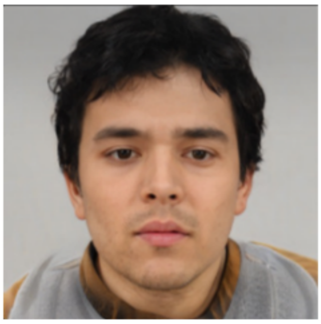	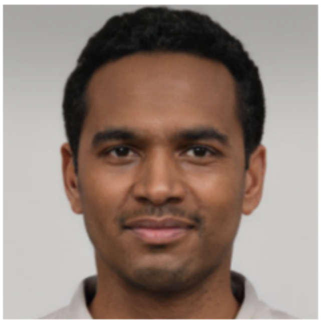	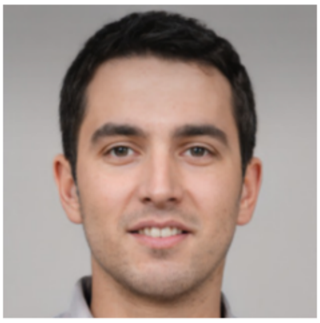
Age: 29<br>Years of experience: 5<br>**A**	Age: 34<br>Years of experience: 2<br>**B**	Age: 40<br>Years of experience: 0<br>**C**

Q3. What factors do you consider when choosing who to hire?

Q4. This space is provided if you have any additional feedback.

(Questionnaire 3 of Study 1A: Isolated Choice condition)

In this study, you will imagine that you are a hiring manager for a foreign trading company. The company is currently trying to hire an Accountant.

It is your goal to hire the candidate whom you think will make the company the most successful.

Here is the job description for the job:

Accountant: An accountant maintains and audits business accounts for the company.

Q1. What's your goal in this survey?

A. Fire an Accountant
B. Evaluate consumer products
C. Hire an Accountant

Q2. There are three candidates for this position. Please choose one form ABC.

Candidates for Accountant

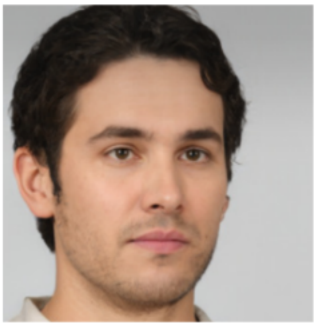

Age: 37
Years of experience: 11
A

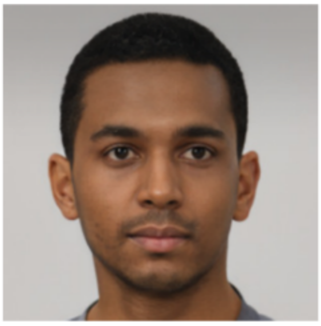

Age: 40
Years of experience: 8
B

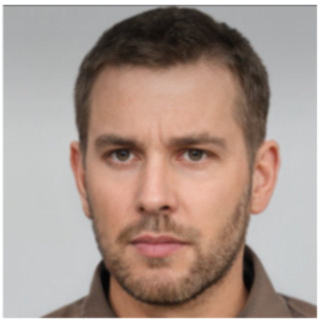

Age: 42
Years of experience: 7
C

Q3. What factors do you consider when choosing who to hire?

Q4. This space is provided if you have any additional feedback.

(Questionnaire 4 of Study 1A: Isolated Choice condition)

In this study, you will imagine that you are a hiring manager for a foreign trading company. The company is currently trying to hire a Quality Inspector.

It is your goal to hire the candidate whom you think will make the company the most successful.

Here is the job description for the job:

Quality Inspector: A quality inspector is responsible for the quality inspection of materials and products.

Q1. What's your goal in this survey?

A. Fire a Quality Inspector
B. Evaluate consumer products
C. Hire a Quality Inspector

Q2. There are three candidates for this position. Please choose one form ABC.

Candidates for Quality Inspector

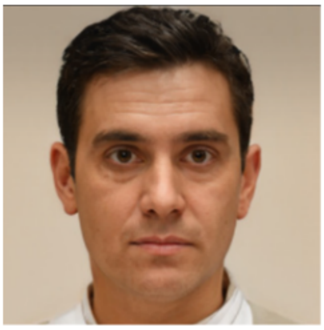

Age: 55
Years of experience: 6
A

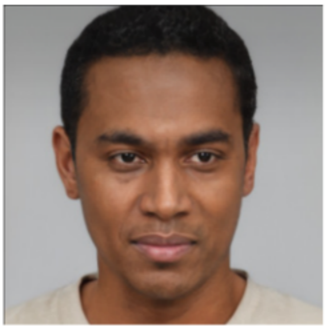

Age: 42
Years of experience: 4
B

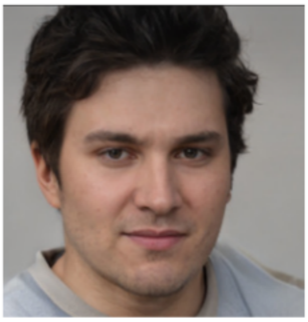

Age: 34
Years of experience: 0
C

Q3. What factors do you consider when choosing who to hire?

Q4. This space is provided if you have any additional feedback.

(Questionnaire 5 of Study 1A: Isolated Choice condition)

In this study, you will imagine that you are a hiring manager for a foreign trading company. The company is currently trying to hire an Administrative Assistant.

It is your goal to hire the candidate whom you think will make the company the most successful.

Here is the job description for the job:

Administrative Assistant: An administrative assistant provides various kinds of administrative support to organization members such as arranging meetings.

Q1. What's your goal in this survey?

A. Fire an Administrative Assistant

B. Evaluate consumer products

C. Hire an Administrative Assistant

Q2. There are three candidates for this position. Please choose one form ABC.

Candidates for Administrative Assistant

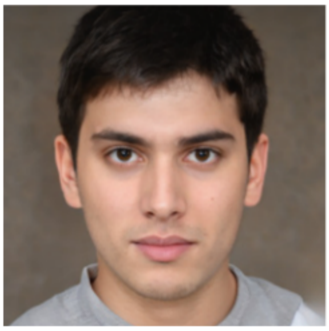

Age: 24
Years of experience: 1
A

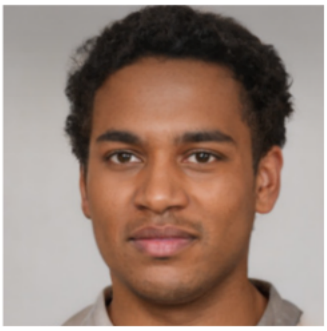

Age: 32
Years of experience: 2
B

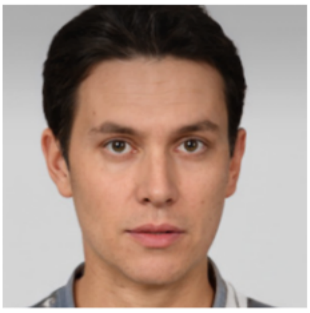

Age: 36
Years of experience: 3
C

Q3. What factors do you consider when choosing who to hire?

Q4. This space is provided if you have any additional feedback.

(Questionnaire 1 of Study 1B: Collective Choice condition)
(The choices for collective choice conditions were shown in a single page in the questionnaires we presented to the participants)

In this study, you will imagine that you are a hiring manager for a restaurant. The restaurant is currently trying to hire five suitable candidates to fill five different positions: Dishwasher, Senior Chef, Kitchen Assistant, Waitress, Restaurant Receptionist.

You need to hire people to fill all five of these positions. It is your goal to hire the candidates whom you think will make the restaurant the most successful.

Here are the job descriptions for each of the job titles:

Dishwasher: A dishwasher is responsible to wash the dishes and dry them.

Senior Chef: A senior chef is the trained professional cook in the restaurant.

Kitchen Assistant: A kitchen assistant is apprenticed to the senior chefs. The kitchen assistant sometimes cook simple cuisines.

Waitress: A waitress brings the food to the customers at their tables.

Restaurant Receptionist: A restaurant receptionist receives customers at the front desk and provides them additional helps.

Q1. What's your goal in this survey?
A. Fire five employees
B. Evaluate consumer products
C. Hire five employees

Q2. There are three candidates for each of the positions. Please choose one from ABC.

Candidates for Dishwasher

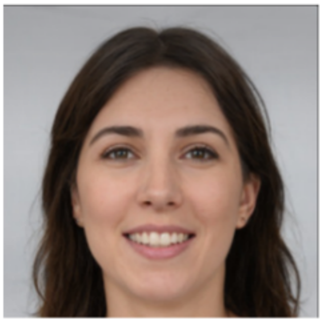

Age: 29
Years of experience: 1
A

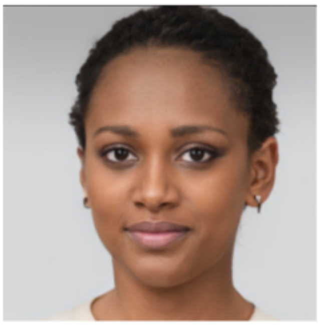

Age: 26
Years of experience: 2
B

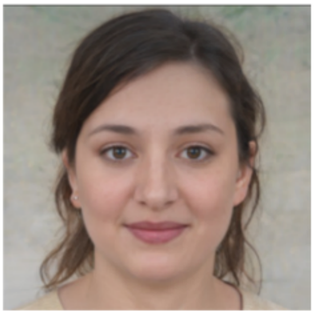

Age: 25
Years of experience: 3
C

Candidates for Kitchen Assistant

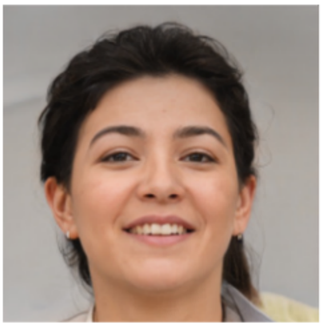

Age: 33
Years of experience: 2
A

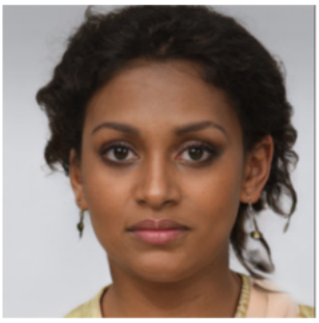

Age: 27
Years of experience: 3
B

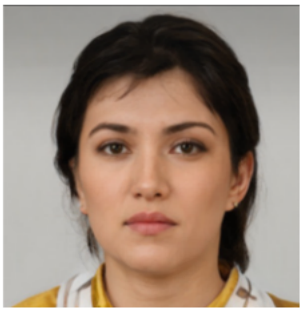

Age: 25
Years of experience: 4
C

Candidates for Senior-Chef

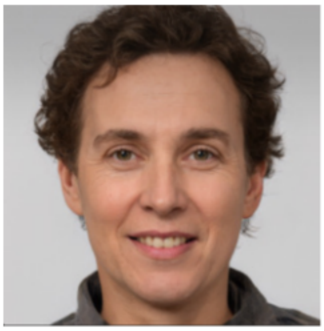

Age: 38
Years of experience: 6
A

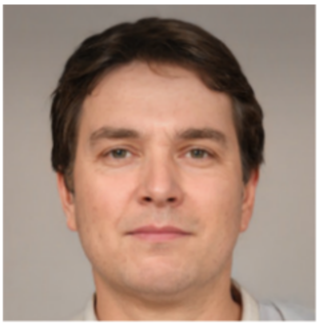

Age: 42
Years of experience: 5
B

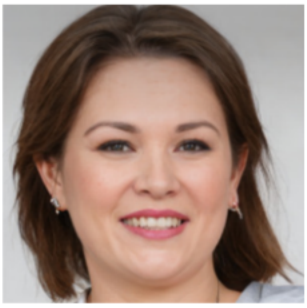

Age: 40
Years of experience: 5
C

Candidates for Waitress

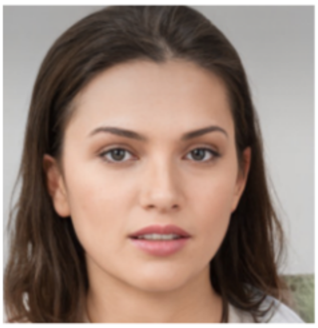

Age: 22
Years of experience: 1
A

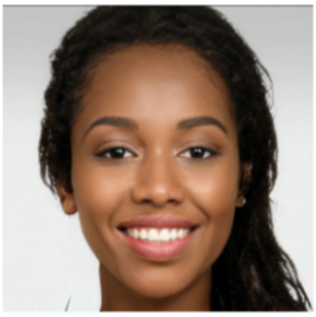

Age: 24
Years of experience: 2
B

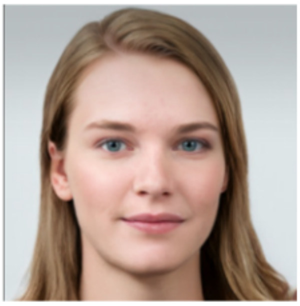

Age: 26
Years of experience: 3
C

Candidates for Restaurant Receptionist

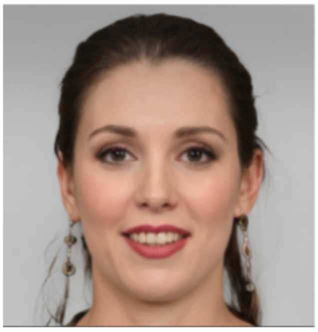

Age: 28
Years of experience: 4
A

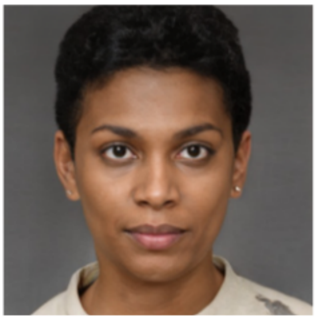

Age: 30
Years of experience: 2
B

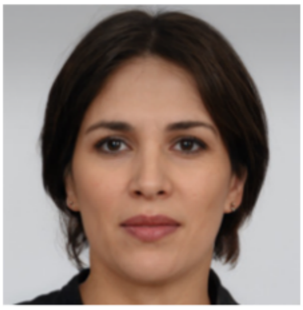

Age: 33
Years of experience: 1
C

Q3. What factors do you consider when choosing who to hire?

Q4. This space is provided if you have any additional feedback.

(Questionnaire 2 of Study 1B: Isolated Choice condition)

In this study, you will imagine that you are a hiring manager for a restaurant. The restaurant is currently trying to hire a Dishwasher.

It is your goal to hire the candidate whom you think will make the restaurant the most successful.

Here is the job description for the job:

Dishwasher: A dishwasher is responsible to wash the dishes and dry them.

Q1. What's your goal in this survey?

A. Fire a Dishwasher
B. Evaluate consumer products
C. Hire a Dishwasher

Q2. There are three candidates for this position. Please choose one form ABC.

Candidates for Dishwasher

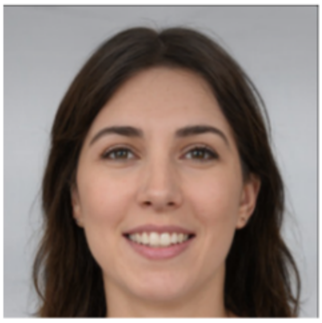

Age: 29
Years of experience: 1
A

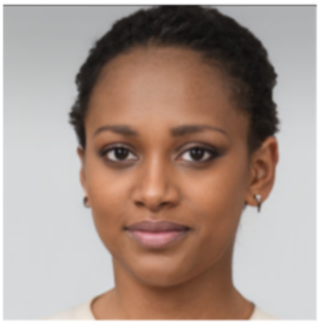

Age: 26
Years of experience: 2
B

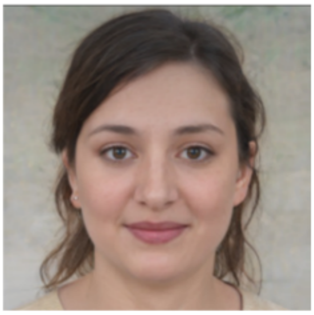

Age: 25
Years of experience: 3
C

Q3. What factors do you consider when choosing who to hire?

Q4. This space is provided if you have any additional feedback.

(Questionnaire 3 of Study 1B: Isolated Choice condition)

In this study, you will imagine that you are a hiring manager for a restaurant. The restaurant is currently trying to hire a Kitchen Assistant.

It is your goal to hire the candidate whom you think will make the restaurant the most successful.

Here is the job description for the job:

Kitchen Assistant: A kitchen assistant is apprenticed to the senior chefs. The kitchen assistant sometimes cook simple cuisines.

Q1. What's your goal in this survey?

A. Fire a Kitchen Assistant
B. Evaluate consumer products
C. Hire a Kitchen Assistant

Q2. There are three candidates for this position. Please choose one form ABC.

Candidates for Kitchen Assistant

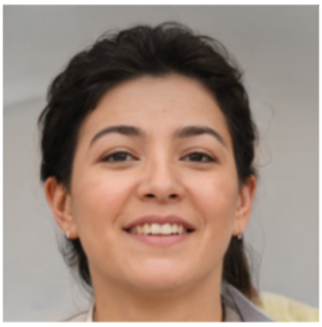

Age: 33
Years of experience: 2
A

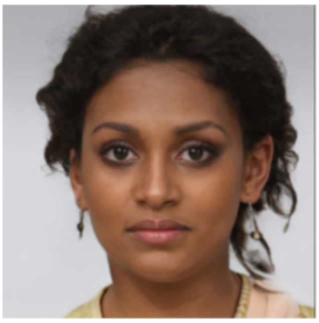

Age: 27
Years of experience: 3
B

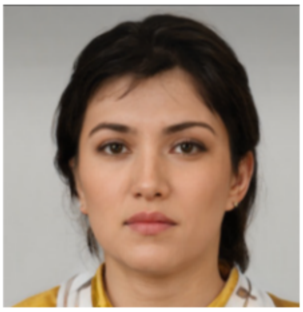

Age: 25
Years of experience: 4
C

Q3. What factors do you consider when choosing who to hire?

Q4. This space is provided if you have any additional feedback.

(Questionnaire 4 of Study 1B: Isolated Choice condition)

In this study, you will imagine that you are a hiring manager for a restaurant. The restaurant is currently trying to hire a Waitress.

It is your goal to hire the candidate whom you think will make the restaurant the most successful.

Here is the job description for the job:

Waitress: A waitress brings the food to the customers at their tables.

Q1. What's your goal in this survey?

A. Fire a Waitress
B. Evaluate consumer products
C. Hire a Waitress

Q2. There are three candidates for this position. Please choose one form ABC.

Candidates for Waitress

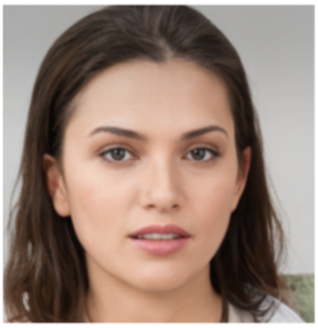

Age: 22
Years of experience: 1
A

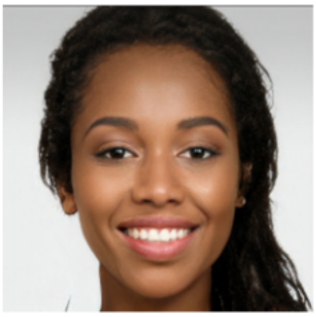

Age: 24
Years of experience: 2
B

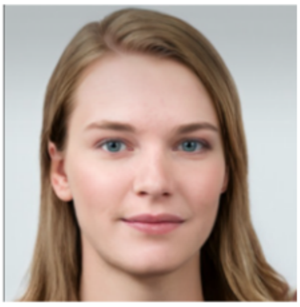

Age: 26
Years of experience: 3
C

Q3. What factors do you consider when choosing who to hire?

Q4. This space is provided if you have any additional feedback.

(Questionnaire 5 of Study 1B: Isolated Choice condition)

In this study, you will imagine that you are a hiring manager for a restaurant. The restaurant is currently trying to hire a Restaurant Receptionist.

It is your goal to hire the candidate whom you think will make the restaurant the most successful.

Here is the job description for the job:

Restaurant Receptionist: A restaurant receptionist receives customers at the front desk and provides them additional helps.

Q1. What's your goal in this survey?

A. Fire a Restaurant Receptionist
B. Evaluate consumer products
C. Hire a Restaurant Receptionist

Q2. There are three candidates for this position. Please choose one form ABC.

Candidates for Restaurant Receptionist

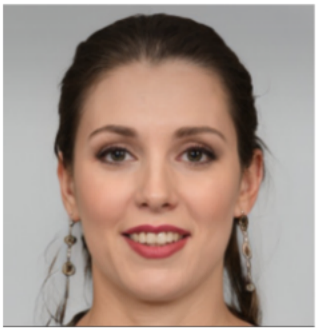

Age: 28
Years of experience: 4
A

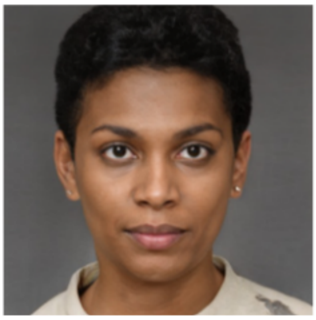

Age: 30
Years of experience: 2
B

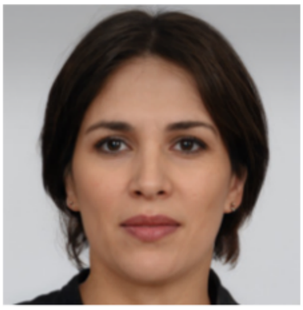

Age: 33
Years of experience: 1
C

Q3. What factors do you consider when choosing who to hire?

Q4. This space is provided if you have any additional feedback.

(Questionnaire 1 of Study 2A: Collective Choice condition)
(The choices for collective choice conditions were shown in a single page in the questionnaires we presented to the participants)

In this study, you will imagine that you are a personnel manager for a foreign trading company. The company is currently trying to lay off five employees in response to economic depression.

You need to fire one people for each position. It is your goal to make decisions to make the company better off.

Here are the job descriptions for each of the job titles:

Purchasing Specialist: A purchasing specialist is responsible for buying the high quality goods at the lowest possible price and in the appropriate quantity.

Accountant: An accountant maintains and audits business accounts for the company.

Area Sales Representative: An area sales representative is responsible for selling the products to consumers in the specific district and ensuring that consumers have positive experiences with the products.

Quality Inspector: A quality inspector is responsible for the quality inspection of materials and products.

Administrative Assistant: An administrative assistant provides various kinds of administrative support to organization members such as arranging meetings.

Q1. What's your goal in this survey?
A. Fire five employees
B. Evaluate consumer products
C. Hire five employees

Q2. There are three employees for each of the positions. Please choose one from ABC. Please make sure that you are firing rather than hiring.

Employees for Purchasing Specialist

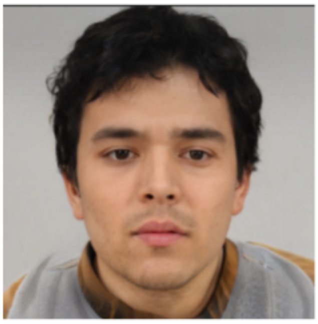

Age: 29
Years of experience: 5
A

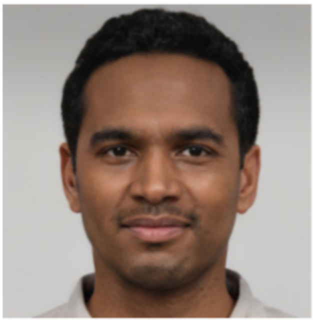

Age: 34
Years of experience: 2
B

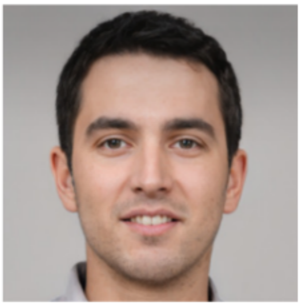

Age: 40
Years of experience: 0
C

Employees for Accountant

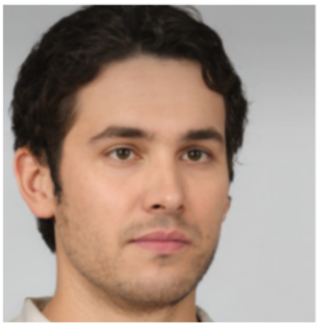

Age: 37
Years of experience: 11
A

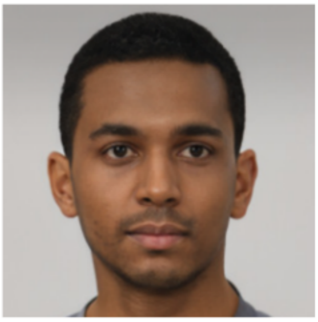

Age: 40
Years of experience: 8
B

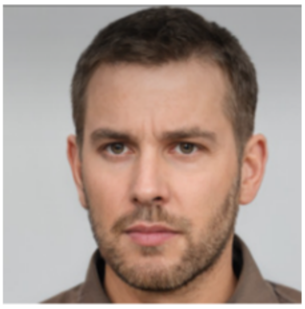

Age: 42
Years of experience: 7
C

Employees for Area Sales Representative

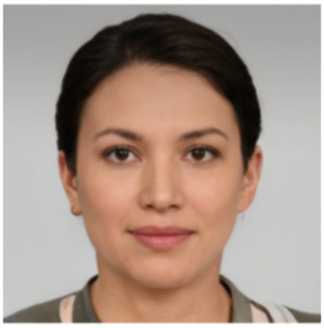

Age: 27
Years of experience: 4
A

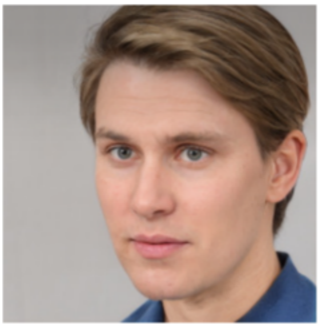

Age: 34
Years of experience: 2
B

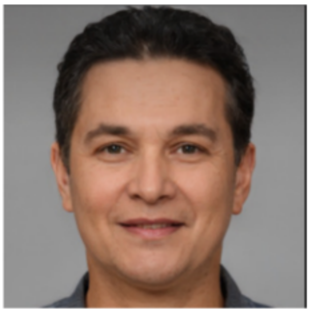

Age: 53
Years of experience: 8
C

Employees for Quality Inspector

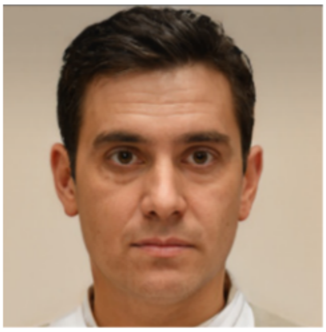

Age: 55
Years of experience: 6
A

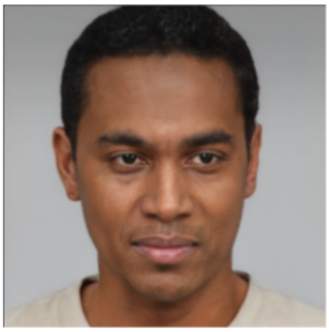

Age: 42
Years of experience: 4
B

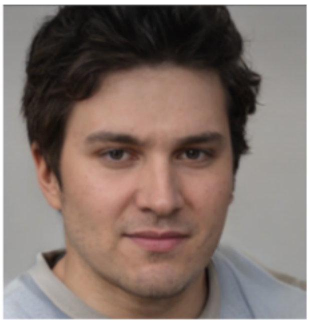

Age: 34
Years of experience: 0
C

Employees for Administrative Assistant

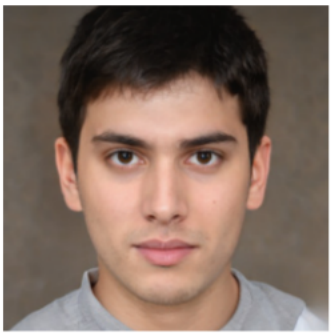

Age: 24
Years of experience: 1
A

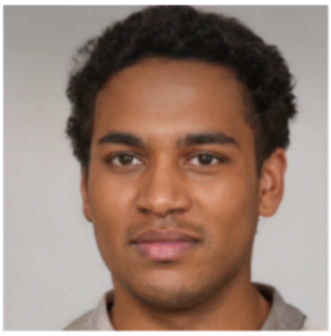

Age: 32
Years of experience: 2
B

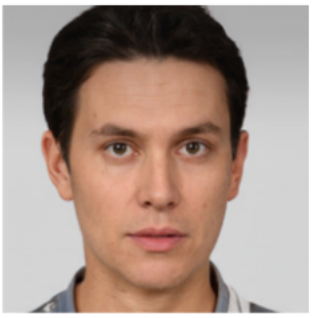

Age: 36
Years of experience: 3
C

Q3. What factors do you consider when choosing who to fire?

Q4. This space is provided if you have any additional feedback.

(Questionnaire 2 of Study 2A: Isolated Choice condition)

In this study, you will imagine that you are a personnel manager for a foreign trading company. The company is currently trying to lay off a Purchasing Specialist in response to economic depression.

It is your goal to make a decision to make the company better off.

Here is the job description for the job title:

Purchasing Specialist: A purchasing specialist is responsible for buying the high quality goods at the lowest possible price and in the appropriate quantity.

Q1. What's your goal in this survey?

A. Fire a Purchasing Specialist
B. Evaluate consumer products
C. Hire a Purchasing Specialist

Q2. There are three employees for this position. Please choose one form ABC. Please make sure that you are firing rather than hiring.

Employees for Purchasing Specialist

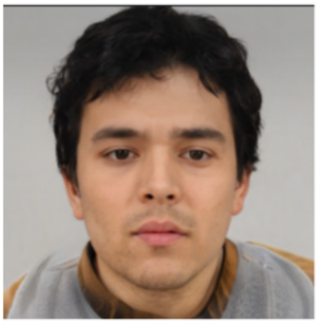

Age: 29
Years of experience: 5
A

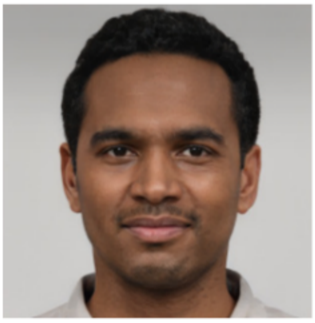

Age: 34
Years of experience: 2
B

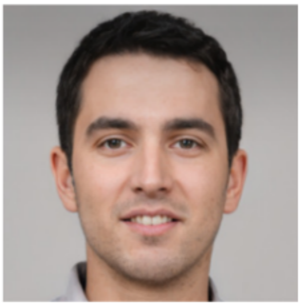

Age: 40
Years of experience: 0
C

Q3. What factors do you consider when choosing who to fire?

Q4. This space is provided if you have any additional feedback.

(Questionnaire 3 of Study 2A: Isolated Choice condition)

In this study, you will imagine that you are a personnel manager for a foreign trading company. The company is currently trying to lay off an Accountant in response to economic depression.

It is your goal to make a decision to make the company better off.

Here is the job description for the job title:

Accountant: An accountant maintains and audits business accounts for the company.

Q1. What's your goal in this survey?
A. Fire an Accountant
B. Evaluate consumer products
C. Hire an Accountant

Q2. There are three employees for this position. Please choose one form ABC. Please make sure that you are firing rather than hiring.

Employees for Accountant

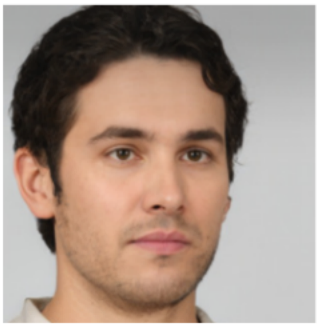

Age: 37
Years of experience: 11
A

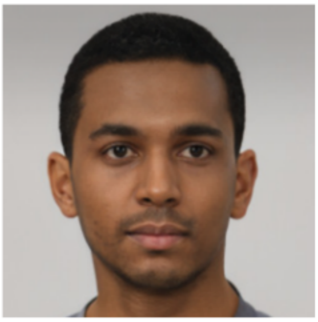

Age: 40
Years of experience: 8
B

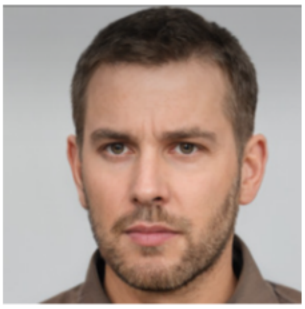

Age: 42
Years of experience: 7
C

Q3. What factors do you consider when choosing who to fire?

Q4. This space is provided if you have any additional feedback.

(Questionnaire 4 of Study 2A: Isolated Choice condition)

In this study, you will imagine that you are a personnel manager for a foreign trading company. The company is currently trying to lay off a Quality Inspector in response to economic depression.

It is your goal to make a decision to make the company better off.

Here is the job description for the job title:

Quality Inspector: A quality inspector is responsible for the quality inspection of materials and products.

Q1. What's your goal in this survey?
A. Fire a Quality Inspector
B. Evaluate consumer products
C. Hire a Quality Inspector

Q2. There are three employees for this position. Please choose one form ABC. Please make sure that you are firing rather than hiring.

Employees for Quality Inspector

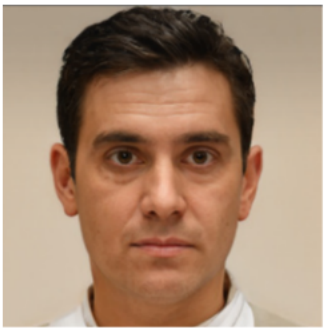

Age: 55
Years of experience: 6
A

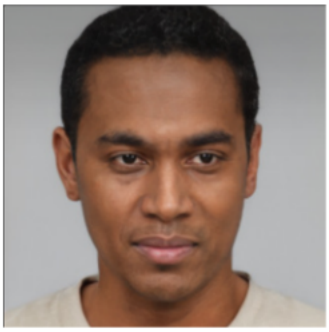

Age: 42
Years of experience: 4
B

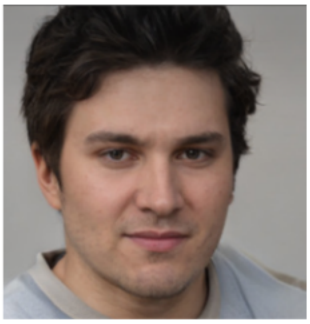

Age: 34
Years of experience: 0
C

Q3. What factors do you consider when choosing who to fire?

Q4. This space is provided if you have any additional feedback.

(Questionnaire 5 of Study 2A: Isolated Choice condition)

In this study, you will imagine that you are a personnel manager for a foreign trading company. The company is currently trying to lay off an Administrative Assistant in response to economic depression.

It is your goal to make a decision to make the company better off.

Here is the job description for the job title:

Administrative Assistant: An administrative assistant provides various kinds of administrative support to organization members such as arranging meetings.

Q1. What's your goal in this survey?

A. Fire an Administrative Assistant

B. Evaluate consumer products

C. Hire an Administrative Assistant

Q2. There are three employees for this position. Please choose one form ABC. Please make sure that you are firing rather than hiring.

Employees for Administrative Assistant

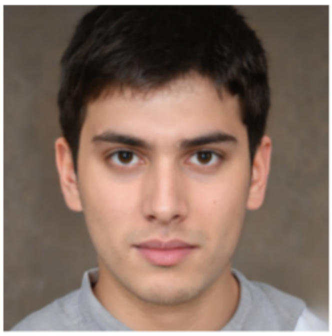

Age: 24
Years of experience: 1
A

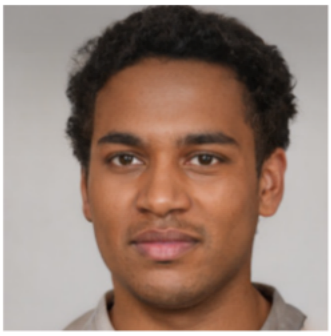

Age: 32
Years of experience: 2
B

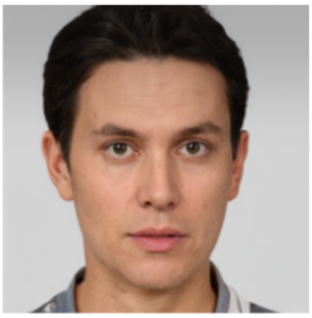

Age: 36
Years of experience: 3
C

Q3. What factors do you consider when choosing who to fire?

Q4. This space is provided if you have any additional feedback.

(Questionnaire 1 of Study 2B: Collective Choice condition)
(The choices for collective choice conditions were shown in a single page in the questionnaires we presented to the participants)

In this study, you will imagine that you are a personnel manager for a restaurant. The restaurant is currently trying to lay off five employees in response to economic depression.

You need to fire one people for each position. It is your goal to make decisions to make the restaurant better off.

Here are the job descriptions for each of the job titles:

Dishwasher: A dishwasher is responsible to wash the dishes and dry them.

Senior Chef: A senior chef is the trained professional cook in the restaurant.

Kitchen Assistant: A kitchen assistant is apprenticed to the senior chefs. The kitchen assistant sometimes cook simple cuisines.

Waitress: A waitress brings the food to the customers at their tables.

Restaurant Receptionist: A restaurant receptionist receives customers at the front desk and provides them additional helps.

Q1. What's your goal in this survey?
A. Fire five employees
B. Evaluate consumer products
C. Hire five employees

Q2. There are three employees for each of the positions. Please choose one from ABC. Please make sure that you are firing rather than hiring.

Employees for Dishwasher

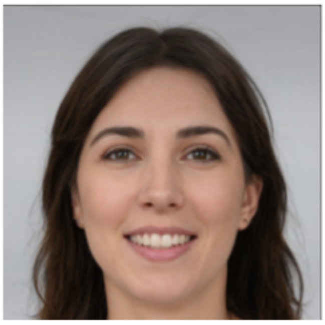

Age: 29
Years of experience: 1
A

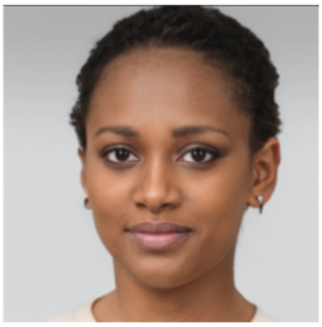

Age: 26
Years of experience: 2
B

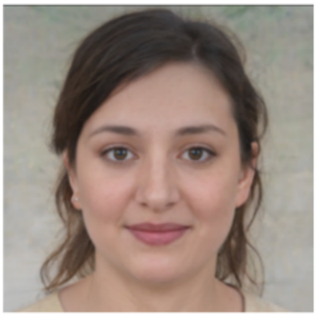

Age: 25
Years of experience: 3
C

Employees for Kitchen Assistant

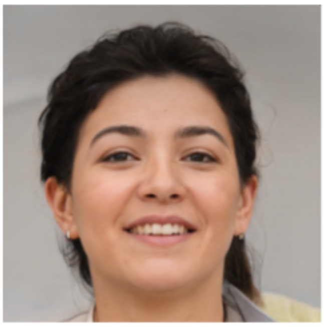

Age: 33
Years of experience: 2
A

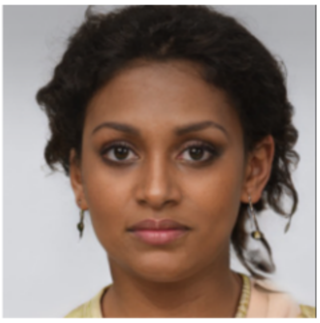

Age: 27
Years of experience: 3
B

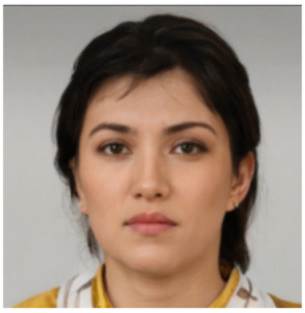

Age: 25
Years of experience: 4
C

Employees for Senior-Chef

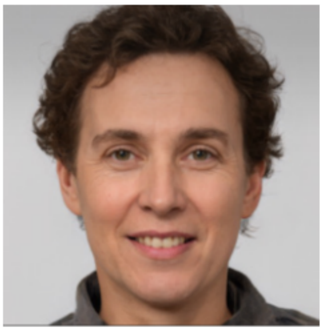

Age: 38
Years of experience: 6
A

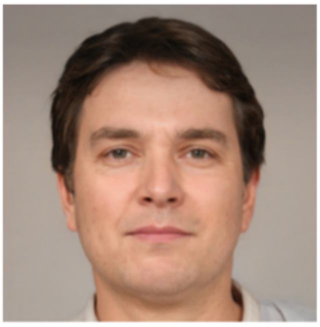

Age: 42
Years of experience: 5
B

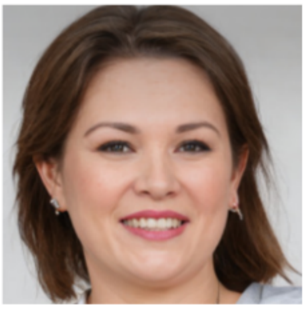

Age: 40
Years of experience: 5
C

Employees for Waitress

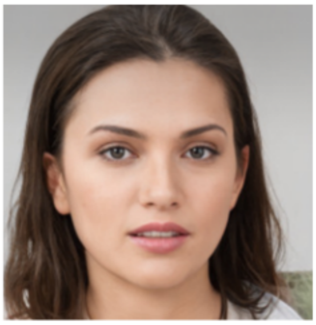

Age: 22
Years of experience: 1
A

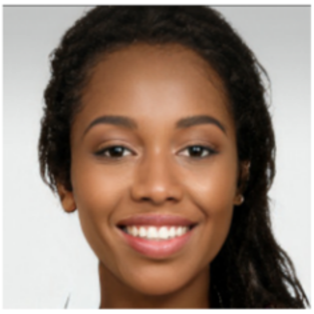

Age: 24
Years of experience: 2
B

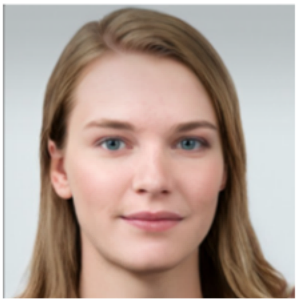

Age: 26
Years of experience: 3
C

Employees for Restaurant Receptionist

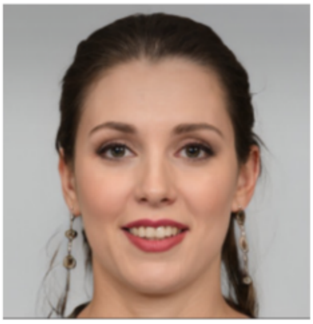

Age: 28
Years of experience: 4
A

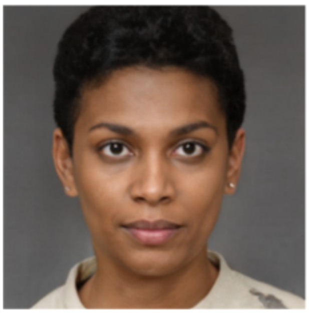

Age: 30
Years of experience: 2
B

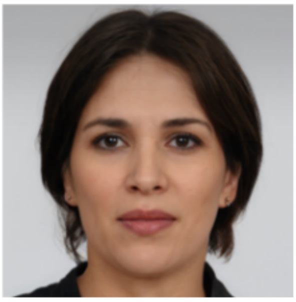

Age: 33
Years of experience: 1
C

Q3. What factors do you consider when choosing who to fire?

Q4. This space is provided if you have any additional feedback.

(Questionnaire 2 of Study 2B: Isolated Choice condition)

In this study, you will imagine that you are a personnel manager for a restaurant. The restaurant is currently trying to lay off a Dishwasher in response to economic depression.

It is your goal to make a decision to make the company better off.

Here is the job description for the job title:

Dishwasher: A dishwasher is responsible to wash the dishes and dry them.

Q1. What's your goal in this survey?
A. Fire a Dishwasher
B. Evaluate consumer products
C. Hire a Dishwasher

Q2. There are three employees for this position. Please choose one form ABC. Please make sure that you are firing rather than hiring.

Employees for Dishwasher

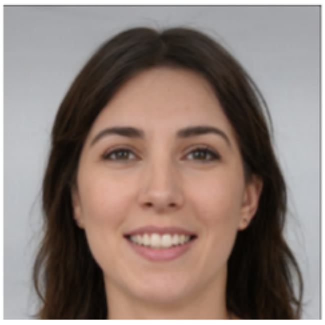

Age: 29
Years of experience: 1
A

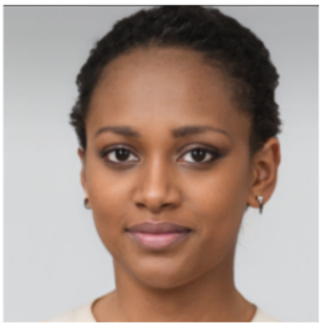

Age: 26
Years of experience: 2
B

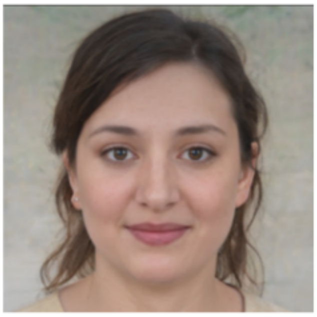

Age: 25
Years of experience: 3
C

Q3. What factors do you consider when choosing who to fire?

Q4. This space is provided if you have any additional feedback.

(Questionnaire 3 of Study 2B: Isolated Choice condition)

In this study, you will imagine that you are a personnel manager for a restaurant. The restaurant is currently trying to lay off a Kitchen Assistant in response to economic depression.

It is your goal to make a decision to make the company better off.

Here is the job description for the job title:

Kitchen Assistant: A kitchen assistant is apprenticed to the senior chefs. The kitchen assistant sometimes cook simple cuisines.

Q1. What's your goal in this survey?
A. Fire a Kitchen Assistant
B. Evaluate consumer products
C. Hire a Kitchen Assistant

Q2. There are three employees for this position. Please choose one form ABC. Please make sure that you are firing rather than hiring.

Employees for Kitchen Assistant

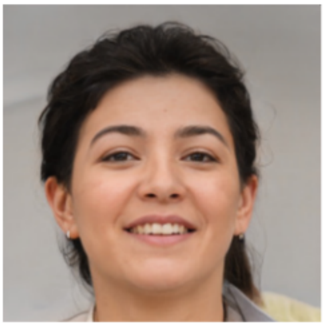

Age: 33
Years of experience: 2
A

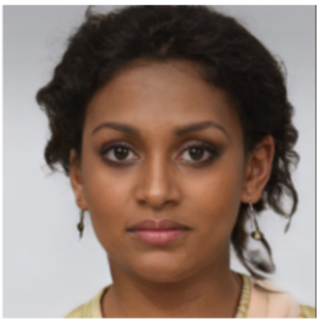

Age: 27
Years of experience: 3
B

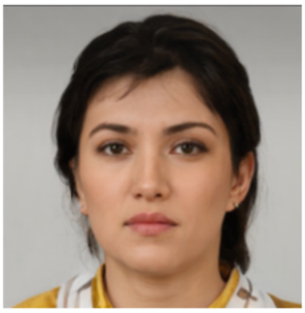

Age: 25
Years of experience: 4
C

Q3. What factors do you consider when choosing who to fire?

Q4. This space is provided if you have any additional feedback.

(Questionnaire 4 of Study 2B: Isolated Choice condition)

In this study, you will imagine that you are a personnel manager for a restaurant. The restaurant is currently trying to lay off a Waitress in response to economic depression.

It is your goal to make a decision to make the company better off.

Here is the job description for the job title:

Waitress: A waitress brings the food to the customers at their tables.

Q1. What's your goal in this survey?
A. Fire a Waitress
B. Evaluate consumer products
C. Hire a Waitress

Q2. There are three employees for this position. Please choose one form ABC. Please make sure that you are firing rather than hiring.

Employees for Waitress

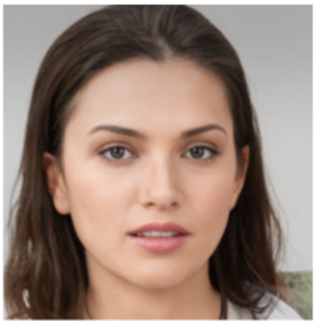

Age: 22
Years of experience: 1
A

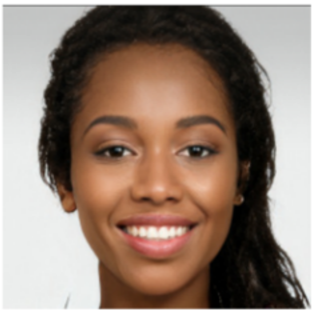

Age: 24
Years of experience: 2
B

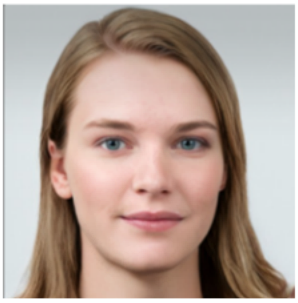

Age: 26
Years of experience: 3
C

Q3. What factors do you consider when choosing who to fire?

Q4. This space is provided if you have any additional feedback.

(Questionnaire 5 of Study 2B: Isolated Choice condition)

In this study, you will imagine that you are a personnel manager for a restaurant. The restaurant is currently trying to lay off a Restaurant Receptionist in response to economic depression.

It is your goal to make a decision to make the company better off.

Here is the job description for the job title:

Restaurant Receptionist: A restaurant receptionist receives customers at the front desk and provides them additional helps.

Q1. What's your goal in this survey?
A. Fire a Restaurant Receptionist
B. Evaluate consumer products
C. Hire a Restaurant Receptionist

Q2. There are three employees for this position. Please choose one form ABC. Please make sure that you are firing rather than hiring.

Employees for Restaurant Receptionist

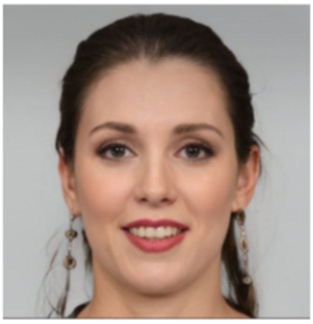

Age: 28
Years of experience: 4
A

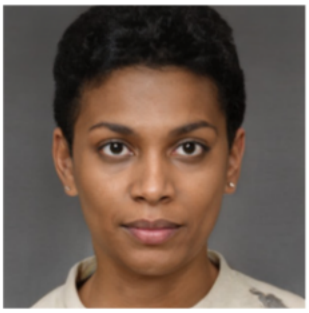

Age: 30
Years of experience: 2
B

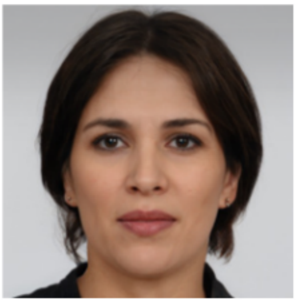

Age: 33
Years of experience: 1
C

Q3. What factors do you consider when choosing who to fire?

Q4. This space is provided if you have any additional feedback.

(Questionnaire 1 of Study 3A: Collective Choice condition)

(The choices for collective choice conditions were shown in a single page in the questionnaires we presented to the participants)

In this study, you will imagine that you are the interviewer of a research institution that is looking for some foreign male researchers. There are three groups of candidates. Please choose one from each group to enter the institution.

It is your goal to select the candidates whom you think are the most eligible.

Q1. How many people do you need to choose in this survey?

A. 1

B. 2

C. 3

Q2. There are five candidates for each group. Please choose one from ABCDE.

Group 1

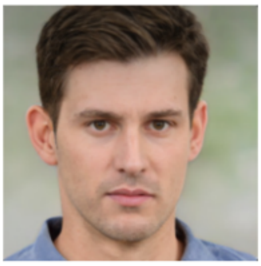

Age: 30
Years of experience: 3
A

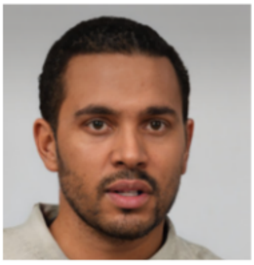

Age: 35
Years of experience: 7
B

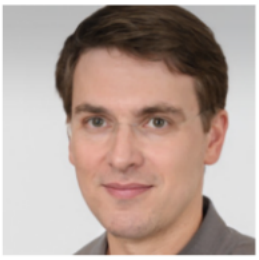

Age: 40
Years of experience: 8
C

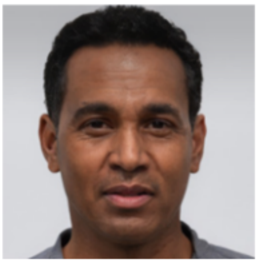

Age: 43
Years of experience: 5
D

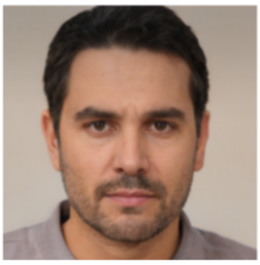

Age: 45
Years of experience: 4
E

Group 2

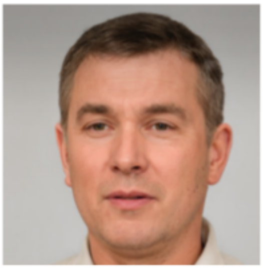

Age: 55
Years of experience: 2
A

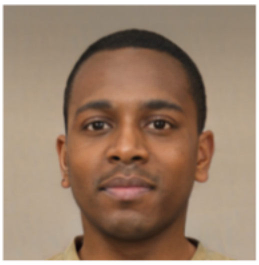

Age: 48
Years of experience: 3
B

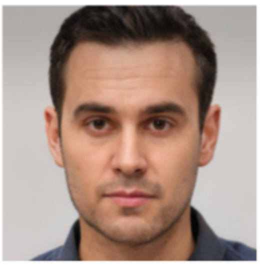

Age: 45
Years of experience: 4
C

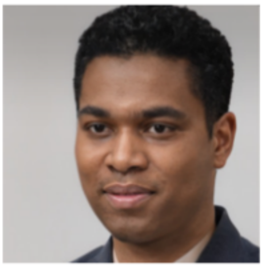

Age: 40
Years of experience: 5
D

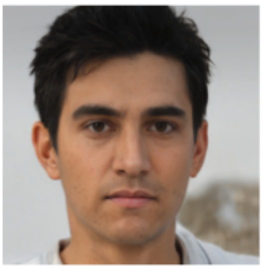

Age: 36
Years of experience: 6
E

Group 3

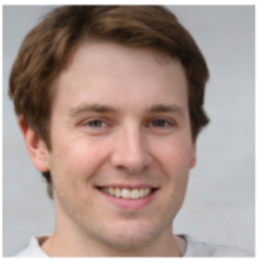

Age: 31
Years of experience: 2
A

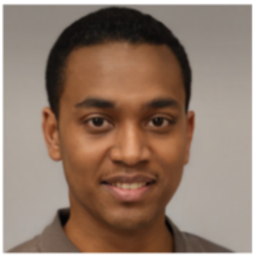

Age: 35
Years of experience: 3
B

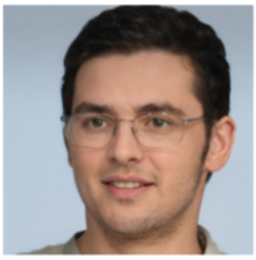

Age: 37
Years of experience: 4
C

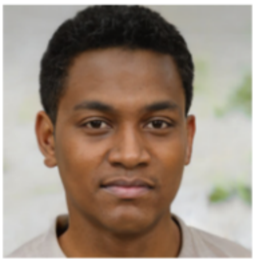

Age: 38
Years of experience: 5
D

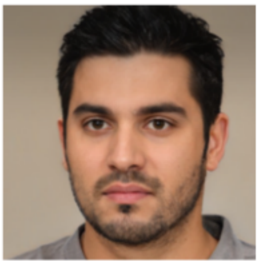

Age: 39
Years of experience: 6
E

Q3. What factors do you consider when choosing who to hire?

Q4. This space is provided if you have any additional feedback.

(Questionnaire 2 of Study 3A: Isolated Choice condition)

In this study, you will imagine that you are the interviewer of a research institution that is looking for a foreign male researcher. Please choose one from the five candidates.

It is your goal to select the candidate whom you think is the most eligible.

Q1. How many people do you need to choose in this survey?

A. 1

B. 2

C. 3

Q2. There are five candidates for the interview. Please choose one from ABCDE.

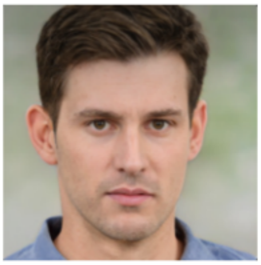

Age: 30
Years of experience: 3
A

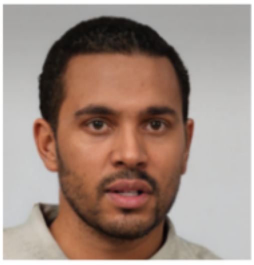

Age: 35
Years of experience: 7
B

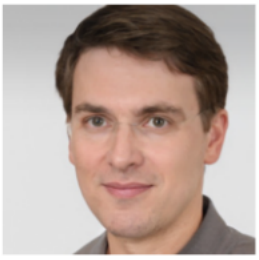

Age: 40
Years of experience: 8
C

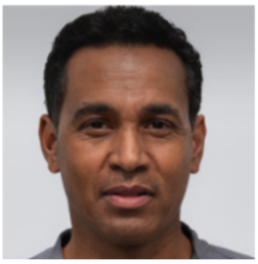

Age: 43
Years of experience: 5
D

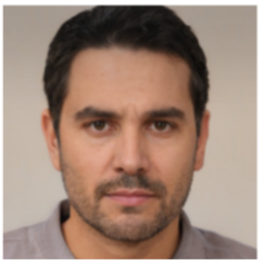

Age: 45
Years of experience: 4
E

Q3. What factors do you consider when choosing who to hire?

Q4. This space is provided if you have any additional feedback.

(Questionnaire 3 of Study 3A: Isolated Choice condition)

In this study, you will imagine that you are the interviewer of a research institution that is looking for a foreign male researcher. Please choose one from the five candidates.

It is your goal to select the candidate whom you think is the most eligible.

Q1. How many people do you need to choose in this survey?

A. 1

B. 2

C. 3

Q2. There are five candidates for the interview. Please choose one from ABCDE.

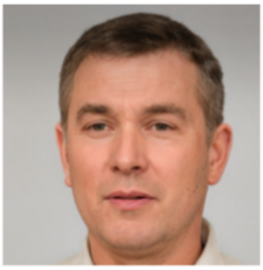

Age: 55
Years of experience: 2
A

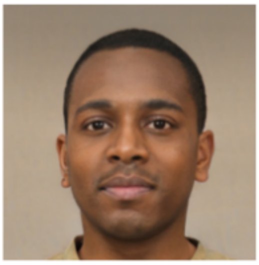

Age: 48
Years of experience: 3
B

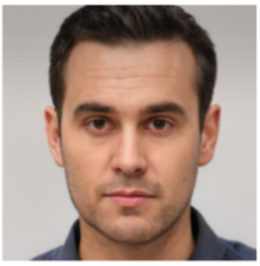

Age: 45
Years of experience: 4
C

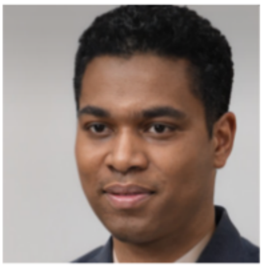

Age: 40
Years of experience: 5
D

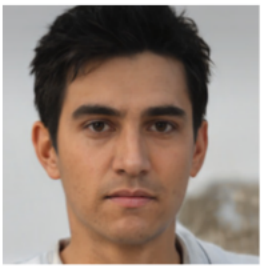

Age: 36
Years of experience: 6
E

Q3. What factors do you consider when choosing who to hire?

Q4. This space is provided if you have any additional feedback.

(Questionnaire 4 of Study 3A: Isolated Choice condition)

In this study, you will imagine that you are the interviewer of a research institution that is looking for a foreign male researcher. Please choose one from the five candidates.

It is your goal to select the candidate whom you think is the most eligible.

Q1. How many people do you need to choose in this survey?

A. 1

B. 2

C. 3

Q2. There are five candidates for the interview. Please choose one from ABCDE.

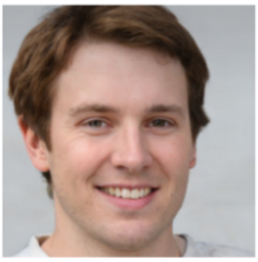

Age: 31
Years of experience: 2
A

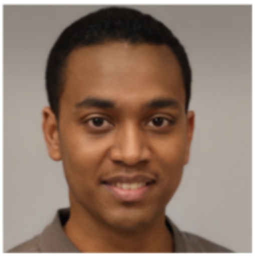

Age: 35
Years of experience: 3
B

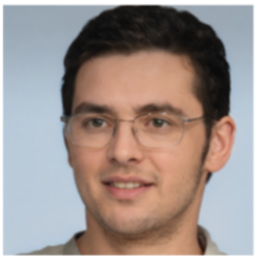

Age: 37
Years of experience: 4
C

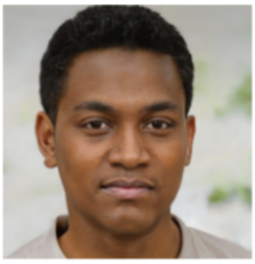

Age: 38
Years of experience: 5
D

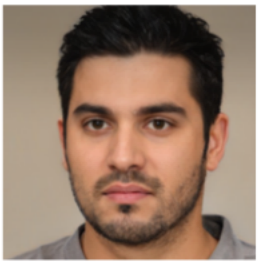

Age: 39
Years of experience: 6
E

Q3. What factors do you consider when choosing who to hire?

Q4. This space is provided if you have any additional feedback.

(Questionnaire 1 of Study 3B: Collective Choice condition)
(The choices for collective choice conditions were shown in a single page in the questionnaires we presented to the participants)

In this study, you will imagine that you are the interviewer of a cram school that is looking for some foreign language teachers. There are three groups of candidates. Please choose one from each group to enter the school.

It is your goal to select the candidates whom you think are the most eligible.

Q1. How many people do you need to choose in this survey?
A. 1
B. 2
C. 3

Q2. There are five candidates for each group. Please choose one from ABCDE.

Group 1

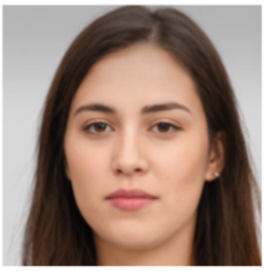

Age: 29
Years of experience: 1
A

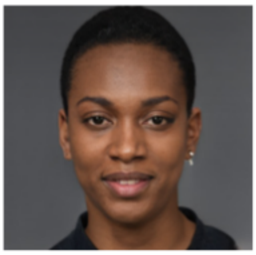

Age: 33
Years of experience: 2
B

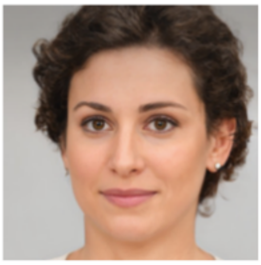

Age: 35
Years of experience: 3
C

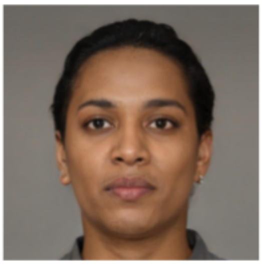

Age: 37
Years of experience: 4
C

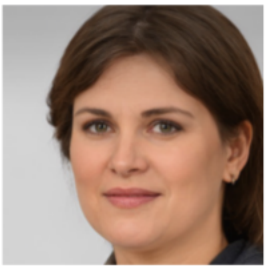

Age: 40
Years of experience: 5
D

Group 2

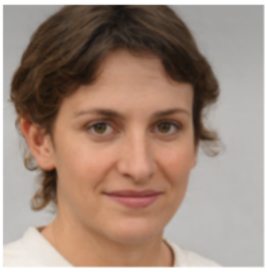

Age: 45
Years of experience: 5
A

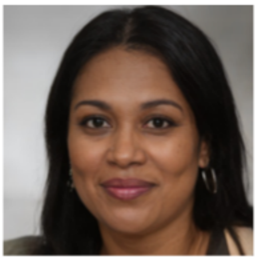

Age: 43
Years of experience: 4
B

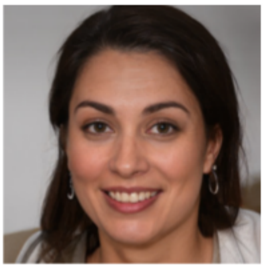

Age: 43
Years of experience: 3
C

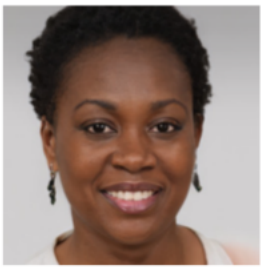

Age: 40
Years of experience: 2
D

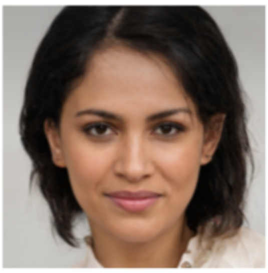

Age: 33
Years of experience: 1
E

Group 3

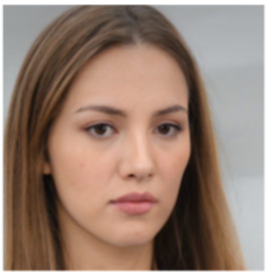

Age: 30
Years of experience: 5
A

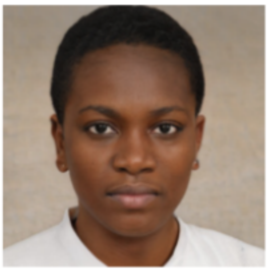

Age: 31
Years of experience: 4
B

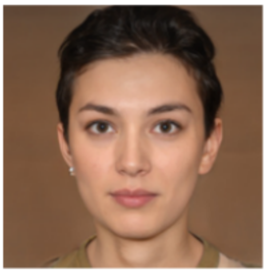

Age: 33
Years of experience: 3
C

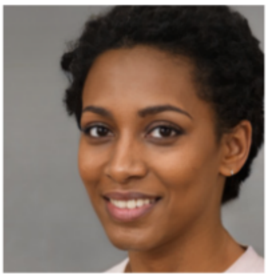

Age: 36
Years of experience: 2
D

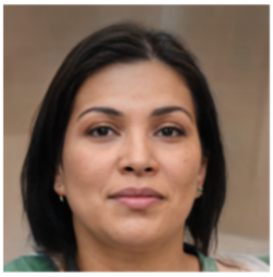

Age: 38
Years of experience: 1
E

Q3. What factors do you consider when choosing who to hire?

Q4. This space is provided if you have any additional feedback.

(Questionnaire 2 of Study 3B: Isolated Choice condition)

In this study, you will imagine that you are the interviewer of a cram school that is looking for a foreign language teacher. There are five candidates. Please choose one to enter the school.

It is your goal to select the candidate whom you think is the most eligible.

Q1. How many people do you need to choose in this survey?

A. 1

B. 2

C. 3

Q2. There are five candidates for the interview. Please choose one from ABCDE.

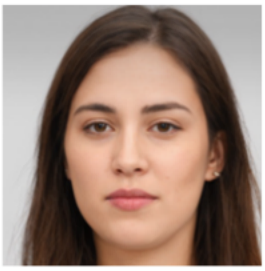

Age: 29
Years of experience: 1
A

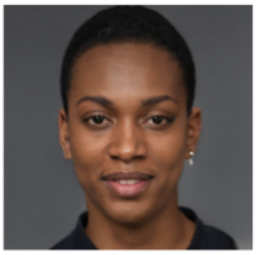

Age: 33
Years of experience: 2
B

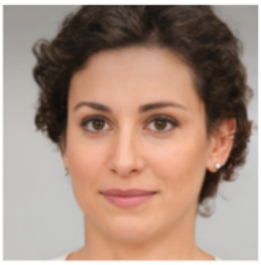

Age: 35
Years of experience: 3
C

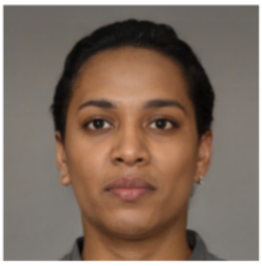

Age: 37
Years of experience: 4
C

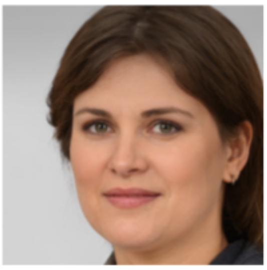

Age: 40
Years of experience: 5
D

Q3. What factors do you consider when choosing who to hire?

Q4. This space is provided if you have any additional feedback.

(Questionnaire 3 of Study 3B: Isolated Choice condition)

In this study, you will imagine that you are the interviewer of a cram school that is looking for a foreign language teacher. There are five candidates. Please choose one to enter the school.

It is your goal to select the candidate whom you think is the most eligible.

Q1. How many people do you need to choose in this survey?

A. 1

B. 2

C. 3

Q2. There are five candidates for the interview. Please choose one from ABCDE.

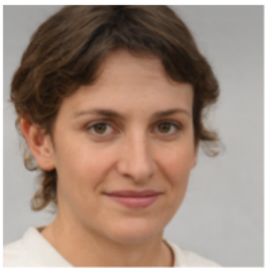

Age: 45
Years of experience: 5
A

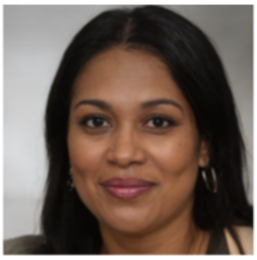

Age: 43
Years of experience: 4
B

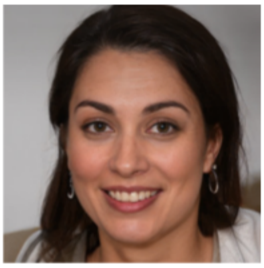

Age: 43
Years of experience: 3
C

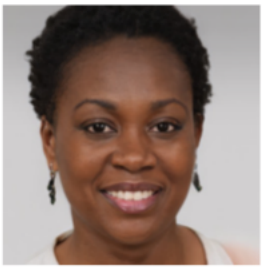

Age: 40
Years of experience: 2
D

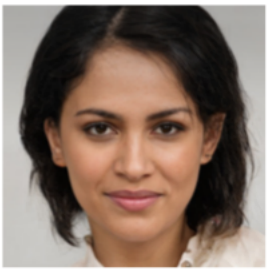

Age: 33
Years of experience: 1
E

Q3. What factors do you consider when choosing who to hire?

Q4. This space is provided if you have any additional feedback.

(Questionnaire 4 of Study 3B: Isolated Choice condition)

In this study, you will imagine that you are the interviewer of a cram school that is looking for a foreign language teacher. There are five candidates. Please choose one to enter the school.

It is your goal to select the candidate whom you think is the most eligible.

Q1. How many people do you need to choose in this survey?

A. 1

B. 2

C. 3

Q2. There are five candidates for the interview. Please choose one from ABCDE.

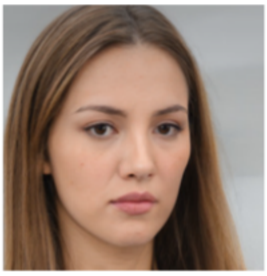

Age: 30
Years of experience: 5
A

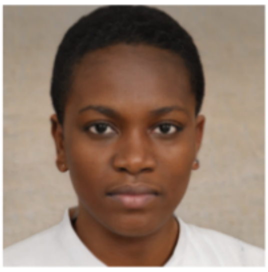

Age: 31
Years of experience: 4
B

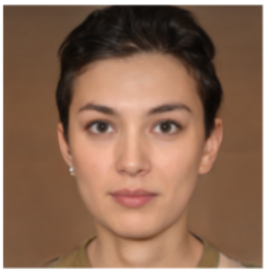

Age: 33
Years of experience: 3
C

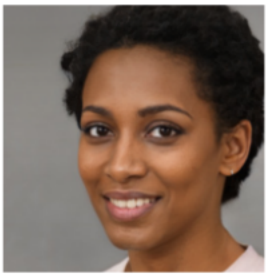

Age: 36
Years of experience: 2
D

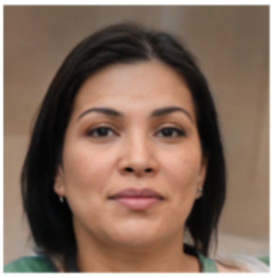

Age: 38
Years of experience: 1
E

Q3. What factors do you consider when choosing who to hire?

Q4. This space is provided if you have any additional feedback.

(Questionnaire 1 of Study 4A: Collective Choice condition)

(The choices for collective choice conditions were shown in a single page in the questionnaires we presented to the participants)

In this study, you will imagine that you are the personnel manager of a translation company. Because of the epidemic, most exhibitions cannot be opened, so the need for interpreters is greatly reduced. The company now is going to lay off several interpreters to cut costs. There are three groups of interpreters. Please lay off one person from each group.

It is your goal to make the firing decisions that will make the company better-off.

Q1. How many people do you need to choose in this survey?

A. 1

B. 2

C. 3

Q2. There are five employees for each group. Please choose one from ABCDE. Please make sure that your are firing rather than hiring.

Group 1

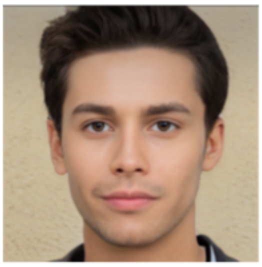

Age: 25
Years of experience: 5
A

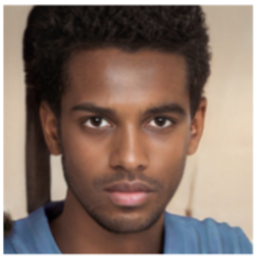

Age: 27
Years of experience: 4
B

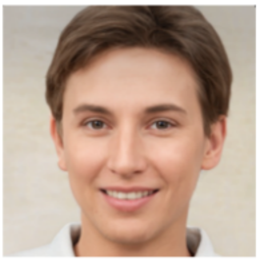

Age: 30
Years of experience: 3
C

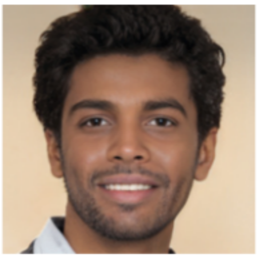

Age: 31
Years of experience: 2
D

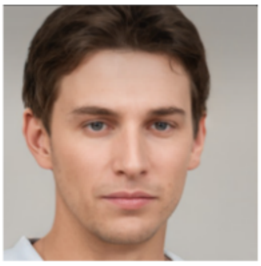

Age: 33
Years of experience: 1
E

Group 2

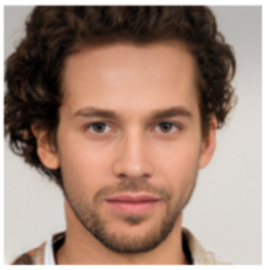

Age: 30
Years of experience: 5
A

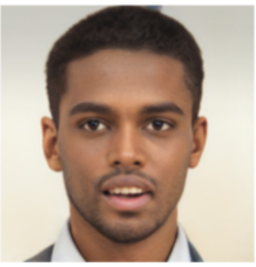

Age: 33
Years of experience: 4
B

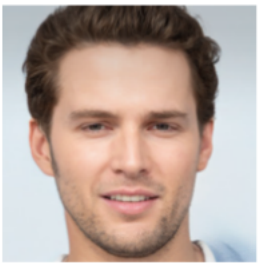

Age: 36
Years of experience: 3
C

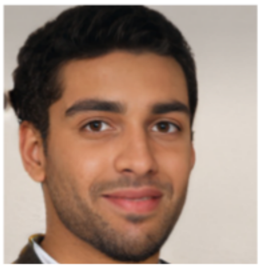

Age: 37
Years of experience: 2
D

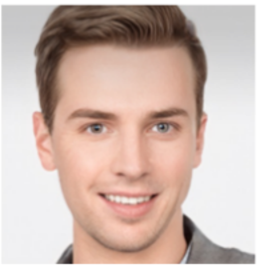

Age: 38
Years of experience: 1
E

Group 3

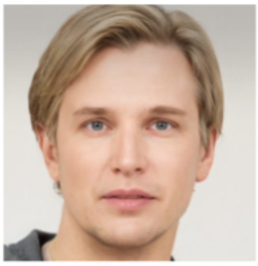

Age: 35
Years of experience: 5
A

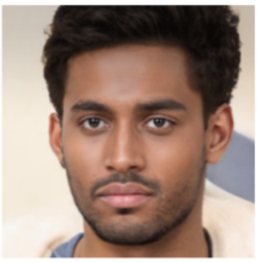

Age: 32
Years of experience: 4
B

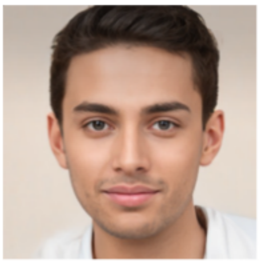

Age: 29
Years of experience: 3
C

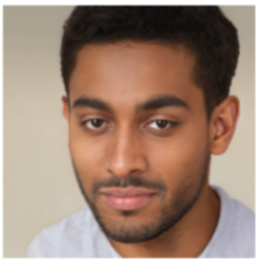

Age: 28
Years of experience: 2
D

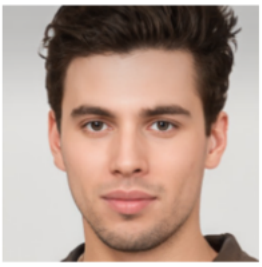

Age: 26
Years of experience: 1
E

Q3. What factors do you consider when choosing who to fire?

Q4. This space is provided if you have any additional feedback.

(Questionnaire 2 of Study 4A: Isolated Choice condition)

In this study, you will imagine that you are the personnel manager of a translation company. Because of the epidemic, most exhibitions cannot be opened, so the need for interpreters is greatly reduced. The company now is going to lay off an interpreter to cut costs. Please lay off one person from five of the employees.

It is your goal to make the firing decision that will make the company better-off.

Q1. How many people do you need to choose in this survey?

A. 1

B. 2

C. 3

Q2. There are five employees. Please choose one from ABCDE. Please make sure that your are firing rather than hiring.

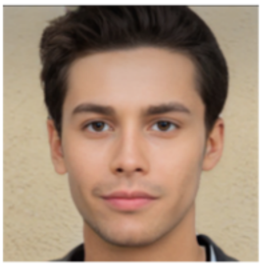

Age: 25
Years of experience: 5
A

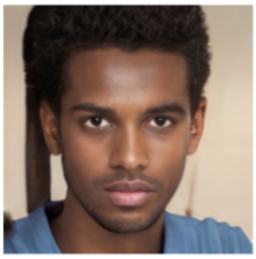

Age: 27
Years of experience: 4
B

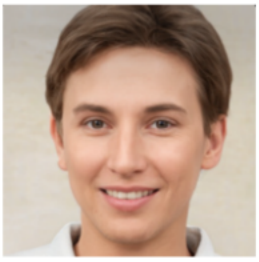

Age: 30
Years of experience: 3
C

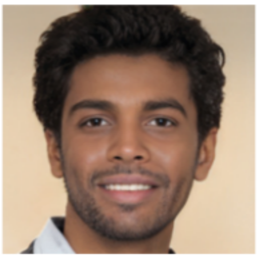

Age: 31
Years of experience: 2
D

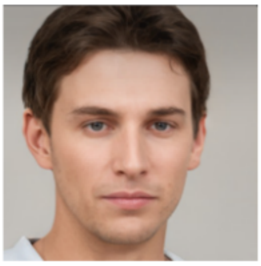

Age: 33
Years of experience: 1
E

Q3. What factors do you consider when choosing who to fire?

Q4. This space is provided if you have any additional feedback.

(Questionnaire 3 of Study 4A: Isolated Choice condition)

In this study, you will imagine that you are the personnel manager of a translation company. Because of the epidemic, most exhibitions cannot be opened, so the need for interpreters is greatly reduced. The company now is going to lay off an interpreter to cut costs. Please lay off one person from five of the employees.

It is your goal to make the firing decision that will make the company better-off.

Q1. How many people do you need to choose in this survey?

A. 1

B. 2

C. 3

Q2. There are five employees. Please choose one from ABCDE. Please make sure that your are firing rather than hiring.

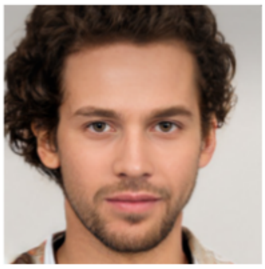

Age: 30
Years of experience: 5
A

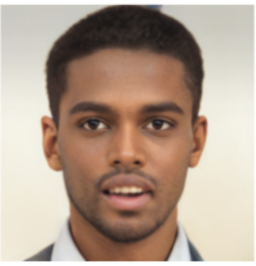

Age: 33
Years of experience: 4
B

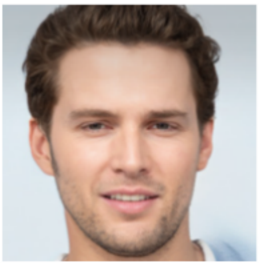

Age: 36
Years of experience: 3
C

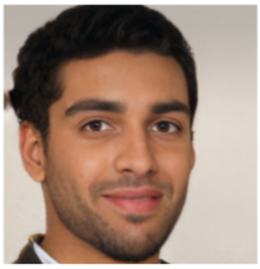

Age: 37
Years of experience: 2
D

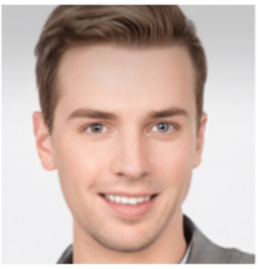

Age: 38
Years of experience: 1
E

Q3. What factors do you consider when choosing who to fire?

Q4. This space is provided if you have any additional feedback.

(Questionnaire 4 of Study 4A: Isolated Choice condition)

In this study, you will imagine that you are the personnel manager of a translation company. Because of the epidemic, most exhibitions cannot be opened, so the need for interpreters is greatly reduced. The company now is going to lay off an interpreter to cut costs. Please lay off one person from five of the employees.

It is your goal to make the firing decision that will make the company better-off.

Q1. How many people do you need to choose in this survey?

A. 1

B. 2

C. 3

Q2. There are five employees. Please choose one from ABCDE. Please make sure that your are firing rather than hiring.

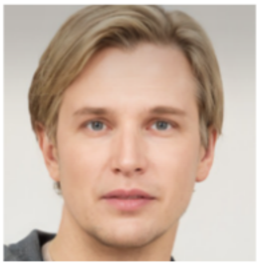

Age: 35
Years of experience: 5
A

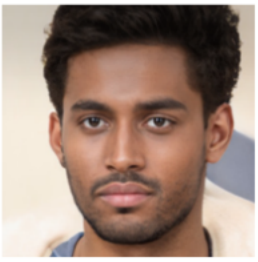

Age: 32
Years of experience: 4
B

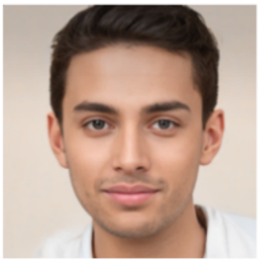

Age: 29
Years of experience: 3
C

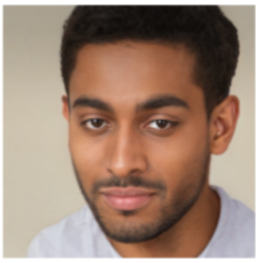

Age: 28
Years of experience: 2
D

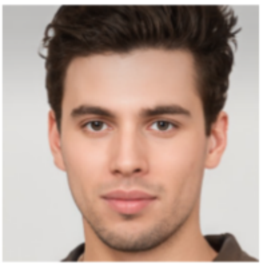

Age: 26
Years of experience: 1
E

Q3. What factors do you consider when choosing who to fire?

Q4. This space is provided if you have any additional feedback.

(Questionnaire 1 of Study 4B: Collective Choice condition)

(The choices for collective choice conditions were shown in a single page in the questionnaires we presented to the participants)

In this study, you will imagine that you are the personnel manager of a translation company. Because of the epidemic, most exhibitions cannot be opened, so the need for interpreters is greatly reduced. The company now is going to lay off several interpreters to cut costs. There are three groups of interpreters. Please lay off one person from each group.

It is your goal to make the firing decisions that will make the company better-off.

Q1. How many people do you need to choose in this survey?

A. 1

B. 2

C. 3

Q2. There are five employees for each group. Please choose one from ABCDE. Please make sure that your are firing rather than hiring.

Group 1

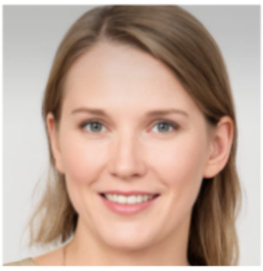

Age: 40
Years of experience: 1
A

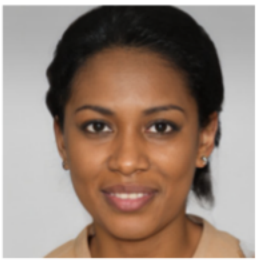

Age: 39
Years of experience: 2
B

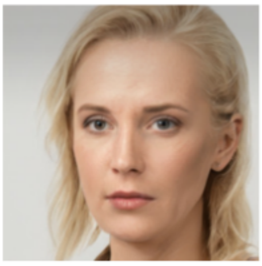

Age: 38
Years of experience: 3
C

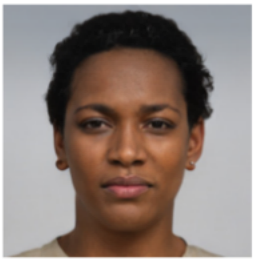

Age: 36
Years of experience: 4
D

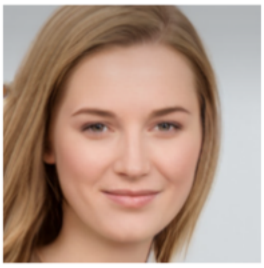

Age: 35
Years of experience: 5
E

Group 2

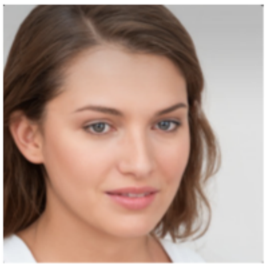

Age: 30
Years of experience: 5
A

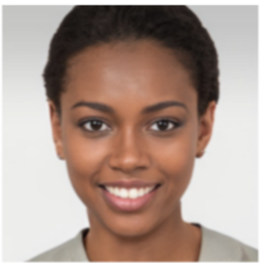

Age: 33
Years of experience: 4
B

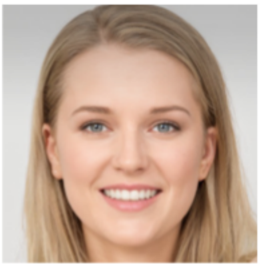

Age: 35
Years of experience: 3
C

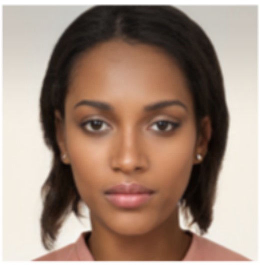

Age: 36
Years of experience: 2
D

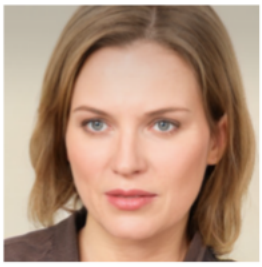

Age: 38
Years of experience: 1
E

Group 3

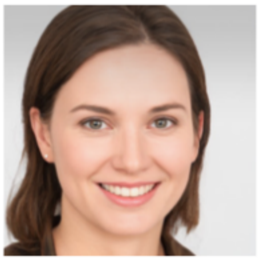

Age: 44
Years of experience: 5
A

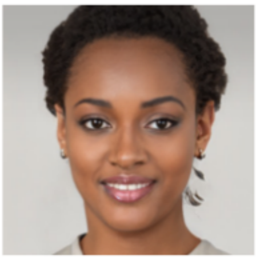

Age: 42
Years of experience: 4
B

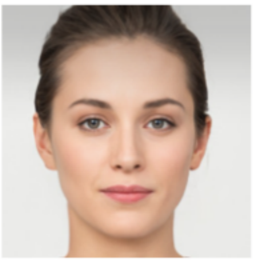

Age: 36
Years of experience: 3
C

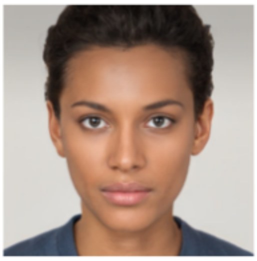

Age: 33
Years of experience: 2
D

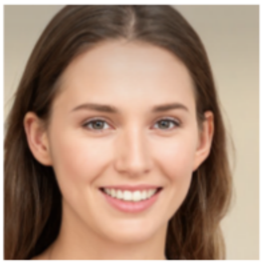

Age: 31
Years of experience: 1
E

Q3. What factors do you consider when choosing who to fire?

Q4. This space is provided if you have any additional feedback.

(Questionnaire 2 of Study 4B: Isolated Choice condition)

In this study, you will imagine that you are the personnel manager of a translation company. Because of the epidemic, most exhibitions cannot be opened, so the need for interpreters is greatly reduced. The company now is going to lay off an interpreter to cut costs. Please lay off one person from five of the employees.

It is your goal to make the firing decision that will make the company better-off.

Q1. How many people do you need to choose in this survey?

A. 1

B. 2

C. 3

Q2. There are five employees. Please choose one from ABCDE. Please make sure that your are firing rather than hiring.

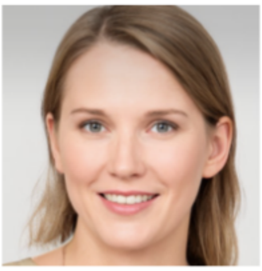

Age: 40
Years of experience: 1
A

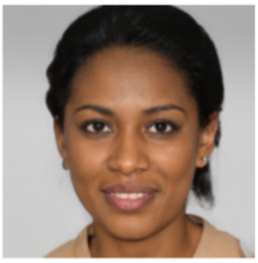

Age: 39
Years of experience: 2
B

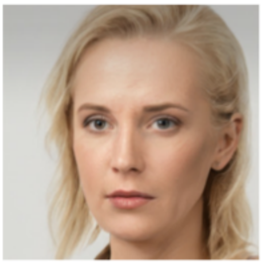

Age: 38
Years of experience: 3
C

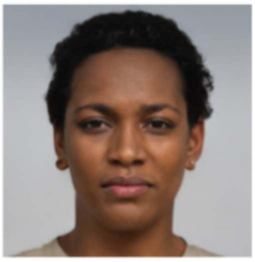

Age: 36
Years of experience: 4
D

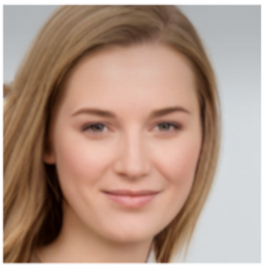

Age: 35
Years of experience: 5
E

Q3. What factors do you consider when choosing who to fire?

Q4. This space is provided if you have any additional feedback.

(Questionnaire 3 of Study 4B: Isolated Choice condition)

In this study, you will imagine that you are the personnel manager of a translation company. Because of the epidemic, most exhibitions cannot be opened, so the need for interpreters is greatly reduced. The company now is going to lay off an interpreter to cut costs. Please lay off one person from five of the employees.

It is your goal to make the firing decision that will make the company better-off.

Q1. How many people do you need to choose in this survey?

A. 1

B. 2

C. 3

Q2. There are five employees. Please choose one from ABCDE. Please make sure that your are firing rather than hiring.

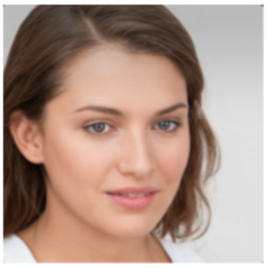

Age: 30
Years of experience: 5
A

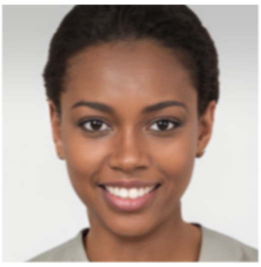

Age: 33
Years of experience: 4
B

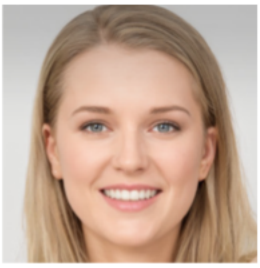

Age: 35
Years of experience: 3
C

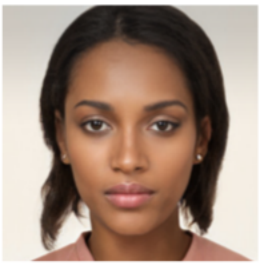

Age: 36
Years of experience: 2
D

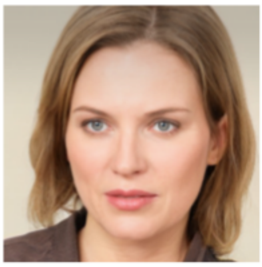

Age: 38
Years of experience: 1
E

Q3. What factors do you consider when choosing who to fire?

Q4. This space is provided if you have any additional feedback.

(Questionnaire 4 of Study 4B: Isolated Choice condition)

In this study, you will imagine that you are the personnel manager of a translation company. Because of the epidemic, most exhibitions cannot be opened, so the need for interpreters is greatly reduced. The company now is going to lay off an interpreter to cut costs. Please lay off one person from five of the employees.

It is your goal to make the firing decision that will make the company better-off.

Q1. How many people do you need to choose in this survey?
A. 1
B. 2
C. 3

Q2. There are five employees. Please choose one from ABCDE. Please make sure that your are firing rather than hiring.

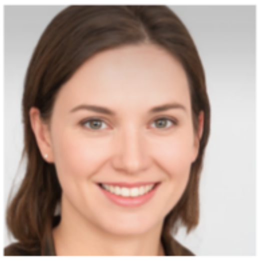

Age: 44
Years of experience: 5
A

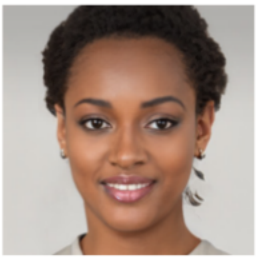

Age: 42
Years of experience: 4
B

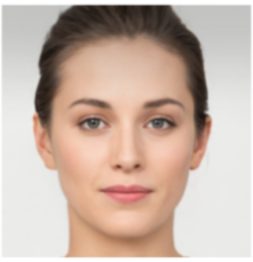

Age: 36
Years of experience: 3
C

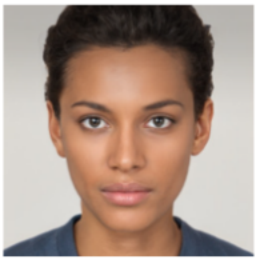

Age: 33
Years of experience: 2
D

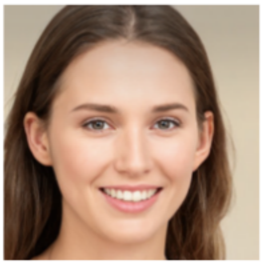

Age: 31
Years of experience: 1
E

Q3. What factors do you consider when choosing who to fire?

Q4. This space is provided if you have any additional feedback.

Human images reproduced with permission from generated.photos

(https://generated.photos/)
